# Identifying molecular tags selectively retained on the surface of brain endothelial cells to generate artificial targets for therapy delivery

**DOI:** 10.1186/s12987-023-00493-6

**Published:** 2023-12-06

**Authors:** Giulia Maria Porro, Italo Lorandi, Xueying Liu, Kazunori Kataoka, Giuseppe Battaglia, Daniel Gonzalez-Carter

**Affiliations:** 1 Institute for Bioengineering of Catalonia (IBEC), Barcelona Institute for Science and Technology (BIST), 08028 Barcelona, Spain; 2 Innovation Center of NanoMedicine (iCONM), Kawasaki Institute of Industrial Promotion, Kawasaki, 210-0821 Japan; 3 Catalan Institution for Research and Advanced Studies (ICREA), 08010 Barcelona, Spain

**Keywords:** Brain targeting, Endocytic rates, Ligand identification, Phage-display, Brain endothelium

## Abstract

Current strategies to identify ligands for brain delivery select candidates based on preferential binding to cell-membrane components (CMC) on brain endothelial cells (EC). However, such strategies generate ligands with inherent brain specificity limitations, as the CMC (e.g., the transferrin receptor TfR1) are also significantly expressed on peripheral EC. Therefore, novel strategies are required to identify molecules allowing increased specificity of therapy brain delivery. Here, we demonstrate that, while individual CMC are shared between brain EC and peripheral EC, their endocytic internalization rate is markedly different. Such differential endocytic rate may be harnessed to identify molecular tags for brain targeting based on their selective retention on the surface of brain EC, thereby generating ‘artificial’ targets specifically on the brain vasculature. By quantifying the retention of labelled proteins on the cell membrane, we measured the general endocytic rate of primary brain EC to be less than half that of primary peripheral (liver and lung) EC. In addition, through bio-panning of phage-displayed peptide libraries, we unbiasedly probed the endocytic rate of individual CMC of liver, lung and brain endothelial cells. We identified phage-displayed peptides which bind to CMC common to all three endothelia phenotypes, but which are preferentially endocytosed into peripheral EC, resulting in selective retention on the surface of brain EC. Furthermore, we demonstrate that the synthesized free-form peptides are capable of generating artificial cell-surface targets for the intracellular delivery of model proteins into brain EC with increasing specificity over time. The developed identification paradigm, therefore, demonstrates that the lower endocytic rate of individual CMC on brain EC can be harnessed to identify peptides capable of generating ‘artificial’ targets for the selective delivery of proteins into the brain vasculature. In addition, our approach identifies brain-targeting peptides which would have been overlooked by conventional identification strategies, thereby increasing the repertoire of candidates to achieve specific therapy brain delivery.

**Supplementary Information:**

The online version contains supplementary material available at 10.1186/s12987-023-00493-6.

## Introduction

Being able to specifically deliver therapies to the brain will greatly benefit the treatment of neurological disorders such as Alzheimer’s disease by increasing the effective therapeutic dose reaching the brain and decreasing detrimental side-effects. To achieve this, therapies (or therapy-loaded vehicles such as nanoparticles) need to selectively interact with, and be taken up by, the brain vasculature with minimal uptake by the vasculature of peripheral organs. With that goal in mind, great efforts have been placed to identify targets on brain endothelial cells (BEC) which promote interaction of therapies with the brain vasculature. To date, identification strategies have focused on target proteins with elevated expression on the surface of BEC [5, 22, 24, 30, 32, 40]. While such an approach has generated important targeting systems to increase brain delivery of therapies [6, 8, 35], these identified ‘natural’ targets have inherent brain-specificity limitations due to significant expression of the target proteins in peripheral tissues, leading to increased off-target accumulation of the carriers in peripheral organs [1, 6, 11, 18, 19, 28, 35].

Therefore, novel strategies are required to direct therapeutic cargo selectively to the brain with minimal increased uptake by the peripheral vasculature. We have previously demonstrated that the lower endocytic rate of BEC, a crucial characteristic of their specialized barrier-forming phenotype [3], may be harnessed to retain antibodies targeting the protein PECAM1 selectively on the surface of BEC [13]. This finding opens up the possibility of exploiting differences in endocytic rates between endothelial phenotypes to identify molecular tags (e.g., peptides or antibodies) which, due to their selective retention on the surface of BEC, generate ‘artificial targets’ on the brain vasculature to achieve therapy delivery specifically to the brain.

However, due to differences in endocytic turn-over of individual proteins arising from function, it is unknown whether this phenomenon would hold true for all cell-membrane components of BEC. Indeed, cell-membrane protein internalization studies have shown the transporter proteins transferrin receptor-1 (TfR1) and low-density lipoprotein receptor (LDLR), two of the principal protein targets employed by brain-delivery systems (e.g., [25, 26, 31, 38]), have the highest rate of endocytic internalization in human brain endothelial cells [15]. Molecular tags binding such proteins, therefore, might be removed preferentially from BEC vs. peripheral endothelial cells (PEC). Hence, strategies aiming to identify molecular tags capable of generating artificial brain targets need to probe the endocytic internalization of individual cell-membrane components with time across different endothelial phenotypes. With that in mind, we have developed an identification paradigm by unbiasedly screening the endocytic internalization rate of cell-membrane components in primary brain and peripheral endothelial cells extracted from rodents to identify peptides selectively retained on the surface of BEC. We demonstrate the identified candidates are able to generate artificial cell-surface targets to promote the intracellular delivery of model proteins with increasing brain-specificity with time. Hence, our approach identifies molecular tags to generate artificial brain targets which would have been overlooked by conventional identification strategies, thereby increasing the repertoire of targeting peptides at our disposal to achieve specific therapy delivery to the brain.

## Results

### Determination of endocytic rates in lung, liver and brain endothelial cells

Among peripheral organs, the liver and lungs significantly contribute to the unspecific and off-target uptake of brain-targeted therapies [13, 35, 36].

**Fig. 1:**
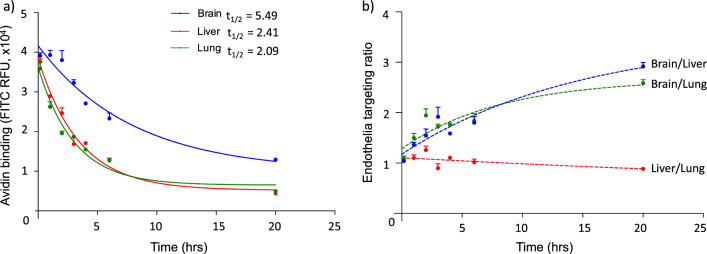
Selective biotin retention on the surface of brain endothelial cells. Biotin tags were placed on the surface of primary endothelial cells derived from rat liver, lung or brain through conjugation of biotin-NHS with primary amines of cell-surface proteins. After incubating cells at 37 °C for varying time-points to allow for the endocytic removal of the biotin tags, the remaining cell-surface biotin was measured through binding of avidin-FITC (**a**). Endothelial targeting by differential retention of cell-surface biotin tags was assessed by the ratio of avidin-FITC binding between different cell types with time (**b**). Results are displayed as mean ± SEM, n = 2 independent experiments (intercalating time-points), triplicate measures. One-phase decay non-linear regression

Hence, the endocytic rates in primary endothelial cells extracted from rat lung, liver or brain were determined by measuring the internalization of cell-membrane proteins over time. To this end, the extracellular domains of cell-surface proteins were molecularly tagged with biotin and their retention on the cell surface after various incubation (37 °C) time-intervals monitored by quantifying avidin (avidin-FITC) binding (Additional file 1: Diagram S1a). The concentration of biotin targets (i.e., biotin-tagged proteins) generated on each endothelial monolayer was comparable between all EC phenotypes, as determined by the similar levels of avidin binding at the initial time-point (t = 0 h, Fig. 1a). Saturation of biotin targets did not account for the comparable levels of avidin binding, since avidin binding increased comparably in all cell types with increasing avidin concentrations (Additional file 1: Fig. S1). However, the retention of biotinylated proteins on the cell-membrane differed markedly between brain EC and peripheral EC. While there was a sharp and comparable decrease in avidin binding to lung and liver EC with time, binding of avidin to brain EC decreased at a markedly lower rate (Fig. 1a). One-phase decay fitting determined the internalization half-life of biotinylated cell-surface proteins on lung, liver and brain EC to be 2.09, 2.41 and 5.49 h, respectively. These results indicate that, while there is a comparable concentration of cell-membrane proteins in all three EC phenotypes, they are retained on the cell-surface of BEC for a markedly longer time. Considering the avidin-biotin interaction as a delivery system, the targeting to each endothelial phenotype can be assessed by their respective avidin-binding ratios (Fig. 1b). While the similar endocytic rates between liver and lung EC resulted in a constant endothelia targeting ratio, the targeting to BEC increased with time against both lung and liver. At the final time-point examined (20 h), brain targeting with respect to both liver and lung increased nearly threefold.

### Identification of molecular tags selectively retained on the surface of BEC

The above results demonstrate the lower rate of endocytosis of cell-surface proteins may be harnessed to increase the targeting of proteins to BEC. To examine if this effect held true for individual cell-surface proteins, we carried out preliminary in vivo experiments testing whether molecular tags (antibodies) binding TfR would similarly be retained on the brain vasculature to enhance brain-targeting of nanoparticles with time (Additional file 1: Fig. S2). TfR was selected as a target since internalization of anti-TfR antibodies has been extensively examined in the past, and TfR represents the most established target for brain delivery [9, 19, 20, 26, 34].

To this end, the biodistribution of avidin-functionalized nanomicelles in mice was assessed as a function of increasing time-intervals between biotinylated anti-TfR antibody and nanomicelle injection. While anti-TfR antibodies increased nanoparticle accumulation more strongly in the brain vs. lungs at short time-points, there was no statistically significant difference at longer time-points (Additional file 1: Fig. S2a). Hence, the brain-to-lung targeting ratio actually decreased with time (Additional file 1: Fig. S2b), indicating the rate of endocytosis of anti-TfR antibodies was faster in brain EC compared to lung EC.

Therefore, to identify molecular tags selectively retained on the surface of BEC by harnessing the differential endocytic profiles of EC, we developed a screening method to unbiasedly probe the endocytic rate of individual cell-membrane components. To this end, we screened a library of phage-displayed peptides against lung, liver or brain endothelial cells to identify two peptide populations in each endothelial phenotype: either peptides with the highest binding to the cell membrane (i.e., the ‘binding population’), or peptides with the lowest endocytic removal from the cell surface (i.e., the ‘retained population’) (Additional file 1: Diagram S2). By comparing the peptide composition of these two populations, and contrasting them to the populations of each endothelia phenotype, we aimed to identify peptides with a long internalization half-life specifically in brain EC.

**Fig. 2:**
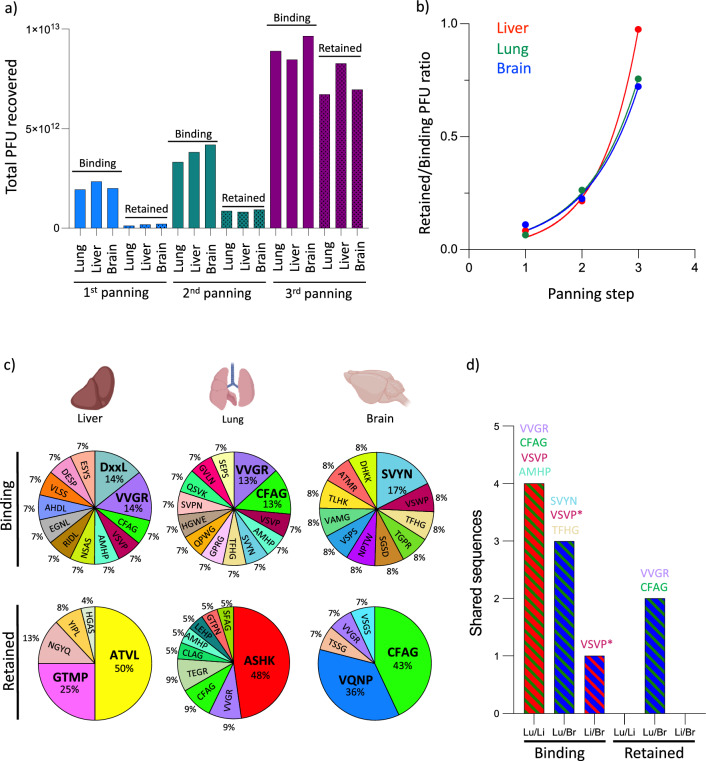
Identification of phage-displayed peptides selectively retained on the surface of endothelial cells. Sequential bio-panning steps of an M13-phage-bound peptide library were carried out on primary endothelial cells derived from rat lung, liver or brain to identify peptides which either bind to each endothelial cell type (binding population), or which bind and are retained on the cell surface after an 8 h time-period (retained population). Peptide recovery after each selection round (bio-panning step) was measured by quantifying phage concentration (PFU) (**a**). Progressive selection of peptides retained on the cell surface was assessed by the ratio of recovered peptides between binding/retained populations after each selection round (**b**). The composition of the final peptide population was assessed by sequencing the DNA of individual phage clones after the third bio-panning step (**c**). Population similarities were assessed by comparing sequences shared between each endothelial phenotype (**d**). *Denotes the VSWP peptide found on brain shared 50% sequence homology with VSVP peptide found on lung and liver

To assess whether the screening procedure enriched phage-displayed peptides preferentially retained on cell surfaces, we firstly quantified the amount of phages recovered from the cell-surface after each bio-panning round (Fig. 2a). Phage recovery was *c.* tenfold lower in the retained population compared to the binding population for all cell types after the first bio-panning round, indicating strong removal of phages from the cell surface. The retained/binding phage ratio of the first round in each EC phenotype (Additional file 1: Fig. S3) mirrored their cell-surface protein endocytic half-lives, with the highest ratio in brain EC, followed by liver and lung EC. Phage recovery increased in the binding population with each bio-panning step, indicating enrichment of phage-displayed peptides which successfully bind to the cell-membrane. Importantly however, phage recovery from the retained population increased even more strongly (Fig. 2a), resulting in an increased ratio of retained/binding phages with each bio-panning round (Fig. 2b). Such increase suggested enrichment of phage-displayed peptides with successful binding to, and slow endocytic removal from the cell-membrane.

To verify the enrichment of phage-displayed peptides by the selection paradigm, we sequenced individual phage clones recovered from the third bio-panning step of each population. In the binding population there was weak peptide enrichment across all endothelia phenotypes, with the most enriched peptides accounting for only 13–17% of the population (Fig. 2c). In addition, there was a strong overlap in the peptide composition between endothelia phenotypes, with a total of 8 individual sequences shared by at least two phenotypes, including the most enriched peptides (Fig. 2d, visualized as Venn diagram in Additional file 1: Fig. S5). The heterogenous peptide composition within individual binding populations and their homogeneity between EC phenotypes suggests a large number of cell-surface components available for peptide binding, which are strongly conserved between endothelial phenotypes.

In contrast, in the retained population there was a strong peptide enrichment across all endothelia phenotypes, with the most enriched peptides accounting for 43–50% of the population (Fig. 2c). In addition, the majority of the enriched peptide sequences were unique to each cell type, with only two shared sequences between phenotypes (Fig. 2d, visualized as Venn diagram in Additional file 1: Fig. S5) (full peptide sequence composition of each population shown on Additional file 1: Fig. S4). These results indicate that while the cell membrane components are highly conserved between endothelial phenotypes, the endocytic rates of individual components have a strong variability both within and between endothelial phenotypes.

Next, we compared individual peptide sequences of the binding and retained populations of each cell type to identify candidates selectively retained on brain endothelial cells. To this end, we focused on sequences enriched in the retained population but not in the binding population of BEC, thereby avoiding retention due to high initial binding (as opposed to slow endocytic removal). Furthermore, we focused on sequences enriched in the binding population of peripheral endothelial cells, but which were not present (or with decreased enrichment) in their retained population, thereby ensuring selection of peptides with slow-endocytic rates specifically in brain EC. One such peptide sequence fulfilled these characteristics: CFAGTPSILMLA (hereafter termed CFAG) was enriched in the binding population of both the liver and lung endothelial cells (7 and 13% population composition, respectively), but disappeared from the former, while it reduced to 9% in the latter in their respective retained populations. Conversely, CFAG had the strongest enrichment in the retained population of brain endothelial cells, while it was not present in the binding population (though its presence in the retained population logically implies that it was present in the initial binding population, but is absent from the final binding population due to competition from peptides with higher cell binding). These results suggest CFAG binds to a cell membrane component which, though present in all three endothelial cell types, has a lower endocytic removal rate from the cell-membrane of brain endothelial cells.

### Generation of artificial targets for protein delivery specifically on brain endothelial cells

Following the identification of phage-displayed peptides selectively retained on the surface of brain endothelial cells, we investigated whether the peptides in their free-form (i.e., synthesized peptides not bound to phage particles) could be employed to generate artificial cell-surface targets for the selective delivery of proteins into brain endothelial cells. To this end, the two most enriched peptide sequences on the retained population of brain EC (i.e., CFAG and VQNP) were synthesized with a biotin conjugate on their N-terminal to deliver avidin as a model protein. As control, we synthesized a reverse sequence of VQNP. As comparison to known brain-targeting ligands, we synthesized a commercially available peptide targeting TfR1 (Tf peptide HAIYPRH) (Additional file 1: Table S1).

**Fig. 3:**
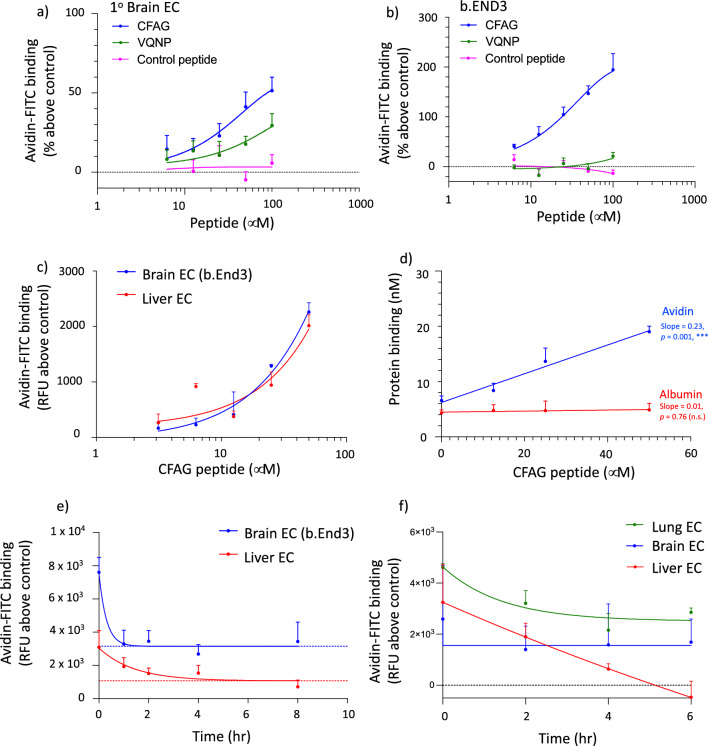
Delivery of avidin-FITC to endothelial cells by biotin-conjugated peptides. Peptides selectively retained on brain endothelial cells were synthesized with biotin conjugated to their N-terminal. Their ability to increase avidin delivery to primary brain endothelial cells (**a**) and b.END3 cells (**b**) was assessed by incubating cells (1 h, 4 °C) with increasing concentrations of peptides followed by incubation (30 min, 4 °C) with avidin-FITC (50 µg/mL) (**a**, **b**). CFAG-biotin-mediated binding of avidin-FITC was similarly compared in primary brain and liver endothelial cells (**c**). The specificity of protein binding was assessed by measuring CFAG-mediated binding of avidin-FITC or albumin-FITC (both at equal molar concentrations) to b.END3 cells (**d**). Internalization rate of CFAG (50 μm) was compared in brain (b.End3) vs. liver EC (**e**) or primary brain EC vs. peripheral EC (**f**) by treatment with avidin-FITC following different time-periods of incubation (37 °C) after CFAG-biotin binding (**e**, dotted line indicates plateau of one-phase decay fitting). Results are displayed as mean ± SEM, of triplicate separate measurements from 3 (**a**, **b**, **e**) or 1 (**c**, **d**, **f**) independent experiments. Slope statistical significance vs. zero value (**c**)

Both CFAG and VQNP efficiently delivered avidin to primary brain endothelial cells (Fig. 3a, Additional file 1: Fig. S6a), demonstrating that the peptides maintained their ability to bind to the endothelial cell surface in their free form, and that they were able to display the biotin conjugate for engaging avidin. Importantly, no avidin delivery was seen by the control peptide (Fig. 3a, Additional file 1: Fig. S6a). Furthermore, the identified peptides achieved higher avidin delivery than the Tf peptide (Additional file 1: Fig. S6c). The delivery of avidin was also examined in brain endothelial cells derived from mice (b.End3 cells). Interestingly, CFAG had a stronger effect on avidin delivery to b.End3, while VQNP failed to increase avidin delivery (Fig. 3b, Additional file 1: Fig. S6b). Similarly to primary brain EC, the control peptide had no effect on avidin delivery while the Tf peptide induced lower avidin delivery than CFAG (Fig. 3b, Additional file 1: Fig. S6b, d). CFAG induced comparable levels of avidin binding to primary brain and liver EC (Fig. 3c), demonstrating lack of intrinsic targeting ability. The specificity of avidin delivery by CFAG was examined by measuring the delivery of albumin as a control protein. No increase in albumin delivery was seen with increasing CFAG concentrations (Fig. 3d), demonstrating the delivery of avidin was specifically due to the protein-biotinylated peptide interaction. In addition, CFAG was able to increase avidin binding to brain endothelial cells in the presence of serum proteins (Additional file 1: Fig. S7a) and had selectivity for brain EC over astrocytes (Additional file 1: Fig. S7b). To test saturation of peptide binding, b.End3 cells were treated with fluorescently-labelled CFAG (Cy5) in the presence of increasing concentrations of unlabelled CFAG (CFAG-biotin). CFAG-Cy5 efficiently bound to the cell surface, but a binding plateau was not reached with the highest concentration tested (100 μm) (Additional file 1: Fig. S7c). Co-treatment with unlabelled CFAG led to a reduction in CFAG-Cy5 binding at the highest concentrations tested (Additional file 1: Fig. S7d).

Next, we examined whether the CFAG peptide in its free form could still exploit the differential endocytic internalization rate of cell-membrane components to generate artificial targets selectively on brain EC. To this end, we measured the internalization dynamics of biotinylated CFAG on brain (b.End3) and liver EC through avidin binding (Additional file 1: Diagram S1a). The half-life of CFAG was shorter in brain EC (0.21 h) vs. liver EC (0.99 h) (Fig. 3e). Interestingly, however, this was due to avidin binding to brain EC decreasing abruptly in the initial hour of incubation, stabilizing thereafter. This effect might be due to serum proteins displacing weakly bound CFAG peptide, akin to the Vroman effect of competitive protein adsorption [17], leaving a population of peptides with higher binding affinity efficiently retained on the cell surface. Hence, despite the shorter half-life, one-phase decay fitting predicted avidin-binding reaching a plateau earlier and at higher avidin levels in brain EC (*c.* 2 h and 3151 RFU, respectively) vs. liver EC (*c.* 6 h and 1068 RFU, respectively). Importantly, the differential endothelia cell-surface retention of CFAG resulted in an increased targeting of avidin to brain EC with time (Additional file 1: Fig. S8a). To corroborate the results obtained with the b.End3 cell line, we measured the CFAG internalization rate employing primary brain endothelial cells, and compared it against both primary lung and liver endothelial cells (Fig. 3f). While CFAG binding was highest for lung > liver > brain EC (mirroring the CFAG enrichment found in the phage ‘binding population’, Fig. 2c), CFAG internalization was slowest for brain EC, followed by lung EC and liver EC (mirroring the CFAG enrichment found in the phage ‘retained population’, Fig. 2c). Similarly to b.End3 cells, there was a fast CFAG internalization into primary BEC within the first two hours of incubation, stabilizing thereafter. Importantly, the differential endocytosis led to an increased targeting of avidin to brain EC vs. peripheral EC with time (Additional file 1: Fig. S8b, c). While the targeting ratio increased sharply in brain vs. liver, the increase in brain vs. lung was less pronounced, indicating differences in endocytic rates of individual cell-membrane components even between peripheral endothelial phenotypes, as can also be seen in the peptide composition of the retention population of liver vs. lung endothelial cells.

### Intracellular protein delivery into brain endothelial cells by identified ligands

**Fig. 4:**
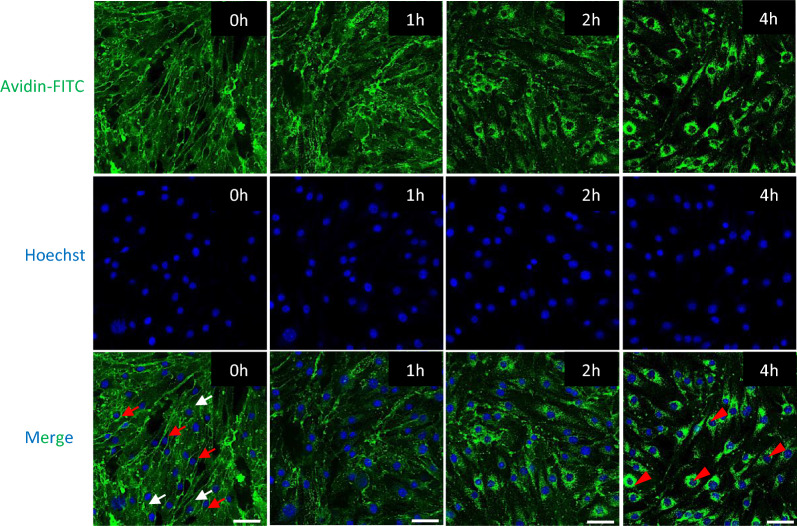
Internalization of proteins mediated by cell-surface bound CFAG peptide. The ability of CFAG peptide (conjugated with N-terminal biotin) to bind and promote internalization of avidin-FITC into b.END3 endothelial cells was examined by labelling their cell surface with CFAG (1 h, 4 °C), followed by incubation with avidin-FITC (30 min, 4 °C). The cells were then incubated at 37 °C for indicated time-points and avidin-FITC localization imaged through confocal microscopy (green signal, FITC; blue signal, Hoechst 33342). Scale bar = 50 µm

The ability of CFAG peptide to generate artificial targets for selective protein delivery to brain EC relies on its low endocytic internalization rate. Therefore, we next examined whether despite its retention on the cell surface, CFAG could induce avidin internalization into brain EC following protein binding. To this end, brain EC (b.End3 cells) were decorated with CFAG peptide and immediately treated with avidin at 4 °C to avoid endocytosis. Protein localization was then visualized at various time-points following incubation at 37 °C (Additional file 1: Diagram S1c) (Fig. 4) (specificity of avidin binding due to presence of biotinylated CFAG peptide was demonstrated by lack of fluorescence signal in the absence of peptide treatment, Additional file 1: Fig. S9). At the initial time-point (0 h), the FITC signal was evenly distributed on the cell surface (as determined by the homogenous distribution in the cell-body areas distant from the nucleus) (white arrows) without penetrating into the cytosol (as determined by strong signal in the inter-cellular contact points and lack of signal in the perinuclear space) (red arrows) (Additional file 1: Diagram S3), indicating avidin bound to CFAG peptide widely distributed on the cell-surface. Within 1 h, the even membrane distribution was disrupted, and a punctate pattern began to appear, indicating endocytic internalization of the avidin-CFAG complex. By 4 h, the cell membrane localization had completely disappeared, with all the FITC signal present in a granular pattern surrounding the nucleus (red arrow heads), indicating successful internalization of the majority of peptide-bound avidin into endocytic vesicles. A similar pattern of avidin-FITC internalization by cell-surface-bound CFAG was seen for primary brain EC (Additional file 1: Fig. S10). Importantly, CFAG was still capable of binding and internalizing avidin-FITC even after a 2 h incubation at 37 °C (Additional file 1: Fig. S11), demonstrating the peptides selectively retained on the surface of brain endothelial cells could still induce protein internalization. Though the extent of internalization was not as pronounced as with the b.End3 cells (or primary brain endothelial cells without prior incubation, Additional file 1: Fig. S10), this could be explained by the decreased levels of CFAG remaining on the cell surface after the initial incubation period.

## Discussion

In the current work we present a novel paradigm to identify molecular tags capable of generating artificial targets to deliver therapies specifically to the brain. Unlike conventional strategies which identify brain-targeting ligands binding to ‘natural’ targets on brain endothelial cells, our strategy identifies molecular tags based on their selective retention on the surface of brain endothelial cells, thereby generating ‘artificial’ targets which can be exploited to deliver cargos specifically to the brain (Additional file 1: Scheme S1).

We have previously demonstrated that the lower endocytic rate of brain endothelial cells can be harnessed to retain antibodies (i.e., molecular tags) against the pan-endothelial protein PECAM-1 selectively on the brain vasculature, thereby targeting nanoparticles to the brain [13]. This effect, however, does not hold true for all cell-membrane components, as the endocytic rate of individual components will vary depending on their function. For instance, proteins transporting essential nutrients across the blood–brain barrier are expected to be internalized at a faster rate than structural proteins. In line with this, we evidenced a higher rate of internalization of anti-TfR antibodies into brain endothelial cells compared to lung endothelial cells, resulting in decreased targeting of nanoparticles to the brain with time. Similarly, a recent proteomic study demonstrated the transporter proteins TfR and LDLR have the highest internalization ratio in human brain endothelial cells [15]. Hence, strategies to identify ligands selectively retained on the brain vasculature need to take into consideration differences in endocytic rates of individual cell membrane components both within individual endothelial cells and between different endothelial phenotypes.

The composition of peptide populations identified through our selection paradigm demonstrates three interesting points: firstly, the cell-membrane of endothelial cells presents a wide-range of targets for peptide binding (as determined by the large number of individual sequences identified in the binding population) which is strongly conserved between different endothelial phenotypes (as determined by the high degree of overlap in shared sequences between phenotypes). In addition, there was higher homology in the cell-membrane composition between peripheral endothelial cells compared to brain endothelial cells, as has been described by ultrastructural studies of glycocalyx composition in peripheral vs. brain capillary endothelial cells [2]. Secondly, despite the similarity in cell-membrane composition, the endocytic rates of individual components differ markedly between endothelial phenotypes, as determined by the low number of shared sequences in the retained population between phenotypes. Thirdly, the strong enrichment of a single peptide sequence in each retained population indicates strong differences in endocytic rates of individual components within each phenotype. Interestingly, the differences in endocytic rates may present a stronger selection pressure for ligand identification than differences in cell-membrane composition. Indeed, the peptide enrichment obtained through this selection paradigm is significantly stronger than the enrichment achieved through selection on differential binding to cell-membrane components of brain endothelial cells [7, 24, 27, 32].

While the cell-membrane components mediating binding of the identified peptides are currently unknown, it is likely they would not be cell-membrane proteins. In line with this, the most abundant and readily available cell-membrane components for peptide binding would be the glycolipids and glycoproteins of the glycocalyx, a protective matrix layer covering the surface of endothelial cells [33]. Binding of the peptides to the glycocalyx to generate ‘artificial’ targets for therapy delivery would have several advantages over binding to specific cell-membrane proteins. Firstly, the steric hinderance experienced by therapies attaching to the ‘artificial’ targets would be minimized, thereby increasing the efficiency of delivery. Steric hinderance by the glycocalyx is known to reduce transport of large molecules into brain endothelial cells by up to 50% [21]. This may explain the higher avidin delivery we observed with the biotinylated CFAG peptide vs. the biotinylated transferrin peptide.

Secondly, peptide binding to the glycocalyx would generate larger numbers of ‘artificial’ targets, as occupancy of the glycocalyx is expected to be saturated at higher concentrations than for specific cell-membrane proteins. As such, adsorptive-mediated transcytosis (AMT), a transport mechanism based on unspecific binding to the cell-surface due to electrostatic interaction, is known to be saturated at concentrations several orders of magnitude higher than receptor-mediated transcytosis (RMT), a transport mechanism based on binding to specific cell-membrane proteins [4, 14], with saturation constants calculated in the micromolar range. Such glycocalyx binding may explain the lack of complete saturation of CFAG peptide binding even at micromolar concentrations. Despite the higher capacity of AMT for particle uptake, it is usually not preferred over RMT due to lack of specificity. Therefore, creating ‘artificial’ targets with a saturation constant similar to AMT, but with an organ distribution restricted to the brain and efficient therapy binding, would have clear advantages over current RMT strategies. Hence, it will be interesting to identify the cell-membrane components mediating retention of peptides on the cell-surface in future studies.

A possible disadvantage of creating ‘artificial’ targets with peptides retained on the cell surface could be lack of cellular internalization. However, delivery systems employing multi-valent carriers could themselves trigger endocytosis through protein clustering or cell-membrane bending [23, 39]. Indeed, we evidenced efficient intracellular delivery of avidin by cell-surface bound CFAG. We attribute this internalization to the tetrameric nature of avidin, being able to multi-valently bind biotin targets to trigger protein clustering and endocytosis.

In conclusion, the molecular tag identification paradigm we present demonstrates cell-membrane components not exclusively present on the brain may be employed to generate ‘artificial’ brain targets by selectively retaining peptides on the surface of brain endothelial cells. This paradigm therefore increases the repertoire of cell membrane components at our disposal for brain targeting and opens the possibility of developing delivery strategies with higher efficiency compared to the current strategies.

## Materials and methods

### Materials and reagents

The phage-displayed peptide library was purchased from New England Biolabs (Ipswich, MA). IPTG, PEG8000, LB broth, tetracycline, agar, fibronectin, collagen, gelatine, bovine fluorescent albumin (albumin-FITC), bovine pancreas trypsin, collagenase type-I, dispase I, and puromycin were purchased from Sigma-Merck (St. Louis, MO). Cell-impermeable biotin-NHS (sulfo-NHS-biotin), deglycosylated avidin (neutravidin)-FITC, and X-gal were purchased from ThermoFisher Scientific (Waltham, MA). Rat primary liver and lung endothelial cells, and peripheral endothelia basal cell culture medium (ECM) were purchased from Cell Biologics (Chicago, IL). Rat primary brain endothelial cells were isolated in-house. Mouse brain endothelial cells b.End3 (CRL-2299) were purchased from the American Type Culture Collection. Microvascular endothelial cell growth medium (EGM-2 plus growth factor supplements) was purchased from Lonza (Basel, CH). All peptides were custom ordered from GenScript Biotech (Piscataway, NJ).

### Cell culturing

Rat primary lung, liver and brain endothelial cells were extracted from the homogenized organs of 6–8 week-old Sprague Dawley rats. Peripheral (lung and liver) endothelial cells were purified by pre-coating cells with anti-PECAM1 (CD31) antibody, followed by separation by secondary antibody-coated magnetic beads (manufacturer’s protocol). Peripheral endothelial cells were plated on gelatin (0.5% w/v)-coated flasks and cultured in endothelial basal medium supplemented with 10% v/v FBS, 1% v/v endothelial cell growth supplements and penicillin (100 IU)/streptomycin (100 µg/mL). Cells were detached by trypsinization and plated on gelatin-coated culture wells. Experimentation was carried out once monolayers reached 100% confluency (2–3 days following plating).

Rat primary brain endothelial cells were extracted as described previously [12, 13]. Briefly, rat brain cortices (cleaned of meninges and visible blood vessels) were homogenized and digested with an enzyme mixture (trypsin, collagenase, dispase). Microvessels were separated from the digested tissue homogenate by centrifugation in a separation gradient buffer (25% v/v BSA). The resulting microvessel pellet was further digested with enzyme mixture and plated in culture flasks coated with collagen/fibronectin. Culturing was done in EGM-2 endothelial cell culture medium (with FBS, VEGF, FGF, IGF, EGF, ascorbic acid, hydrocortisone, gentamycin) (termed full-EGM), supplemented with puromycin (5 days @ 4 µg/mL, 3 days @ 1 µg/mL) to eliminate contaminating cells. Once a pure culture was obtained, cells were detached by trypsinization and plated on collagen/fibronectin-coated wells in full-EGM. Once cells reached 90% confluency, VEGF was removed from the culturing medium to promote a BBB phenotype by reducing para/transcellular permeability [10, 29, 37]. Experimentation was carried out once monolayers reached 100% confluency (2–3 days following removal of VEGF).

Mouse b.End3 cells were detached by trypsinization, plated in collagen-coated cell culture wells in DMEM (10% v/v FBS, penicillin (100 IU)/streptomycin (100 µg/mL)) until reaching 100% confluency (4–5 days).

All cells were maintained at 37 °C in a humidified atmosphere with 5% CO_2_.

### Measurement of endocytic internalization of biotinylated cell-surface proteins

To determine endocytic internalization rates, confluent monolayers of primary rat liver, lung or brain endothelial cells were treated with the cell-membrane impermeable biotinylating reagent sulfo-biotin-NHS (20 min, 4 °C, 500 µg/mL), thereby attaching a biotin molecule to primary amines of the extracellular domain of cell-membrane proteins. Following thorough washing, the cells were incubated in their respective culture medium (37 °C) for appropriate time-periods, after which the cells were washed (HBSS) and treated with neutravidin-FITC (30 min, 4 °C, 50 µg/mL). Neutravidin, a deglycosylated, neutrally charged form of avidin, was employed to minimize unspecific interaction with cell-membrane components [16]. Cells were thoroughly washed (HBSS) to remove unbound neutravidin-FITC and fluorescence (490/525 em/ex) read with a Spark multimode microplate reader (Tecan).

### Phage-displayed peptide selection

Bio-panning selection was carried out on the M13-phage combinatorial library displaying 10^9^ unique dodecamer peptide sequences. Confluent primary rat lung, liver or brain endothelial cells were treated with phages at an initial concentration of 10^11^ pfu/mL (i.e., with 100 copies of each peptide sequence) in HBSS (1 h, 4 °C). After thoroughly washing with HBSS, cells were either incubated in 100 mM citric acid (pH 2.2) to recover cell-surface phages (recovered phages were then neutralized with equal volumes of 1 M Tris-HCl, pH 7.5) or incubated in corresponding cell culture medium (at 37 °C) for 8 h to allow phage endocytic internalization. After this time-point, phages retained on the cell surface were recovered as above. Recovered phages were amplified in *E. coli* as per the manufacturer’s instructions and employed for the subsequent bio-panning steps. The phage purity/concentration of the amplified phage eluate recovered after each bio-panning step was assessed through UV–vis (Additional file 1: Fig. S12).

Unamplified phages from the final bio-panning step were titered in LB-agar plates supplemented with IPTG/X-gal and the DNA from individual plaques derived from library phages (i.e., blue coloured plaques) sequenced to determine their peptide composition, as per the manufacturer’s instructions. In brief, the individual phage plaque was amplified in *E. coli* and the phages isolated through PEG/NaCl precipitation. DNA was isolated by incubating phages in Tris-HCl/EDTA/NaI buffer followed by precipitation in ethanol. The DNA pellet was resuspended in MilliQ H_2_O. Purified ssDNA was quantified by fluorimetry (Qubit ssDNA assay with a Qubit 4 fluorometer) (Invitrogen), and sequenced through Sanger sequencing using the -96 gIII sequencing primer (5′-CCCTCATAGTTAGCGTAACG-3′) performed with BigDye™ Terminator v3.1 Cycle Sequencing Kit and electrophoresed on an Applied Biosystems Automated 3730xl DNA analyzer.

### Peptide synthesis

All peptides were custom synthesized by GenScript Biotech. Either biotin or Cy5 were conjugated onto the peptide N-terminal. In order to recreate the peptide conformation adopted when bound to the phage, the amino acid linker sequence Gly–Gly–Gly–Ser found between the pIII phage protein and the peptide sequence was added to the C-terminal of the 12-amio acid sequence (X_12_GGGS). In addition, the C-terminal was amidated to avoid a negatively charged carboxylate group not found in the phage-bound form. Peptide purity was > 98%.

### Peptide cell-binding

Confluent endothelial monolayers were treated with increasing CFAG-Cy5 concentrations in HBSS (1 h, 4 °C). Cells were thoroughly washed with HBSS and fluorescence (649/667 em/ex) quantified with a Spark multimode microplate reader (Tecan). For competition assays, the same protocol was followed, except cells were incubated with CFAG-Cy5 in the presence of increasing concentrations of unlabelled CFAG (CFAG-biotin).

### Peptide-mediated protein cell delivery

Confluent endothelial monolayers were treated with increasing peptide concentrations in HBSS (1 h at 4 °C, unless otherwise stated). After removing unbound peptide, cells were either incubated in appropriate cell culture medium (37 °C) for varying time-points, or directly treated with neutravidin-FITC or albumin-FITC (0.83 mM, in HBSS, 30 min at 4 °C). After removing unbound protein with thorough HBSS washes, fluorescence (490/525 em/ex) was quantified with a Spark multimode microplate reader (Tecan) or imaged through confocal microscopy (Additional file 1: Diagram S1a–c).

### Nanomicelle synthesis

Nanomicelles were assembled from oppositely charged block copolymers consisting of a poly(ethylene glycol) (PEG) (2.2 k) segment tandemly coupled to either an anionic polypeptide [poly(α,β-aspartic acid)] or a cationic polypeptide [poly(5-aminopentyl-α,β-aspartamide)] (average degree of polymerization = 75) segment. A reactive azide (N_3_) group was attached to the α end of the PEG segment of anionic block copolymers (hereafter, N_3_-anions) to allow functionalization of DBCO-linked proteins through Click chemistry. A fraction of cationic block copolymers were labelled with a Cy5 fluorophore onto the ω end of the polypeptide chain (hereafter, Cy5-cation) to allow for nanomicelle quantification and imaging. Copolymers were blended in 10 mM phosphate buffer (PB; pH 7.4) (N_3_-anion:Cy5-cation:untagged-cation volume ratio = 10:4:16 at 1 mg/mL) to promote self-assembly of nanomicelles with a hydrophilic, noncharged PEG shell with azide reactive group-surface decoration, and Cy5 fluorescence at the core of the nanomicelle. The nanomicelle structure was then stabilized by cross-linking cationic and anionic polypeptide segments with 1-ethyl-3-(3-dimethylaminopropyl) carbodiimide (EDC; 15 h, room temperature), followed by washing through Vivaspin filters (100 kDa molecular mass cutoff [MMCO], polyethersulfone [PES] membrane) (Sartorius) with 10 mM PB (pH 7.4) to remove EDC.

Deglycosylated tetrameric avidin (Neutravidin, 60 kDa molecular mass, FITC-labelled) (ThermoFisher) was reacted with maleimide-PEG4-DBCO (Mal-PEG4-DBCO) (×10 molar excess Mal-PEG4-DBCO) in 10 mM PB at pH 8.5 (15 h, room temperature) to promote binding of maleimide onto primary amines on the protein. Unreacted Mal-PEG4-DBCO was removed by dialysis (3.6 kDa MMCO, PES membrane) against 10 mM PB (pH 7.4), with 200 mM arginine (10 mM PB/Arg) to prevent protein aggregation.

DBCO-linked avidin was then attached to N_3_-decorated nanomicelles by mixing at a 5:1 (protein:nanomicelle) molar ratio in 10 mM PB/Arg (15 h, room temperature). Unreacted avidin proteins were removed by washing through Vivaspin filters (100 kDa MMCO) with 10 mM PB/Arg. To remove contaminating avidin aggregates, avidin-NMs were filtered through a 0.2-µm PES filter (Millipore) with Dulbecco’s PBS (D-PBS) without calcium or magnesium. Avidin-NMs were diluted to a concentration of 1 mg/mL (polymer weight) and kept in D-PBS to allow for direct in vivo injection.

### In vivo TfR-targeting experiments

Balb-c mice (female, 5 wk old) were systemically (tail-vein) injected with D-PBS or biotin-α-TfR (monoclonal rat anti-mouse, 25 µg; Invitrogen). Following appropriate time intervals, the mice were systemically (tail-vein) injected with avidin-NM (200 µg in D-PBS). After 16 h, the mice were anesthetized (isofluorane) and perfused with D-PBS (transhepatic perfusion followed by transcardial perfusion to ensure complete removal of free nanomicelles from vascular lumen) before organ collection into D-PBS and weighing. Organs were then homogenized in a multibead shocker homogenizer (Yasui Kikai) in passive lysis buffer (Promega), followed by nanomicelle fluorescence (Cy5) quantification in the homogenates (containing microvessels) with an Infinite M1000 Pro fluorescence microplate reader (Tecan). Nanomicelle accumulation in antibody-treated animals was quantified as a percentage of nanomicelle accumulation in D-PBS-treated animals.

### Statistical analysis

Statistical analysis was carried out through linear and non-linear (one-phase decay) regression analysis, one-way ANOVA (with Tukey’s *post-hoc* tests), and *t-*tests (unpaired, two-way) for indicated experiments with the use of GraphPad Prism software. Error bars for targeting data (binding ratios) were determined through error propagation calculations from primary data.

### Supplementary Information

**Additional file 1: Figure S1.** Binding of avidin-FITC to biotin-labelled extracellular protein domains on endothelial cells. The extracellular domain of cell-membrane proteins on primary rat lung, liver or brain endothelial cells was biotinylated by conjugation with cell-impermeable biotin-NHS (20 mins, 4 °C). Biotin labelling was then assessed by quantifying binding of avidin-FITC (30 min, 4 °C) at increasing concentrations. Results are displayed as mean ± SEM of triplicate measurements. **Figure S2.** Organ accumulation of nanomicelles functionalized with TfR1-targeting ligands. Mice were injected with biotinylated a-TfR1 antibody (25 μg, tail vein injection). After 15 min or 8 h, mice (two separate groups, each n = 4) were injected with avidin-functionalized polymeric nanomicelles (200 μg, tail vein injection). After 16 h, mice were perfused with PBS and nanomicelle biodistribution quantified in organ homogenates (a). Brain targeting ratio at each time-point is calculated by the ratio of nanomicelle uptake (b). Results are displayed as mean ± SEM,*denotes *p* ≤ 0.05 as determined by a student’s t-test between respective pairs. **Figure S3.** Ratio of phages recovered from the‘retained population’ to the phages recovered from the ‘binding population’ from the first bio-panning round from each endothelial cell type. **Figure S4.** Full DNA/amino acid sequence and frequency of analysed individual clones from the third bio-panning round for each endothelial cell type and selection regime. **Figure S5.** Venn diagram of enriched phage-displayed peptide sequences shared between endothelial phenotypes in both the binding population and the retained population. **Figure S6.** Avidin-FITC binding to 1°BEC (a, c) or b.END3 (b, d) mediated by CFAG-biotin, VQNP-biotin or control peptide-biotin (100 μM) (a, b), or the transferrin peptide HAIYPRH-biotin (c, d). **Figure S7.** Avidin-FITC binding to 1°BEC (a) or C6 astrocytes (b) following incubation with biotinylated peptides (100 μM) in serum containing medium (1 h, 37 °C) (a) or HBSS (1 h, 4  C) (b), respectively. CFAG-Cy5 binding to b.End3 cells at increasing concentrations (c). Competition of CFAG-Cy5 (3.13 μM) peptide binding to b.End3 cells by unlabelled CFAG (CFAG-biotin) (d). **Figure S8.** Endothelial targeting of avidin by differential retention of CFAG on the cell surface was assessed by the ratio of avidin-FITC binding between each cell phenotype with time (a, b). Due to the negative value of avidin-FITC RFU at 6hrs for liver EC in (b) (i.e., avidin-FITC binding was slightly lower compared to binding to cells without CFAG treatment), the brain/liver ratio could not be calculated for this time-point. Hence, we have also displayed the liver/brain and lung/brain targeting ratio (c). **Figure S9.** Avidin-FITC binding to b.End3 cells in the absence (PBS treatment, a) or presence (50 μM, b) of biotinylated CFAG peptide. **Figure S10.** Visualization of avidin-FITC internalization into primary brain endothelial cells mediated by CFAG-biotin (50 μM). Cells were treated with CFAG-biotin (1 h, 4 °C), followed by binding of avidin-FITC (30 mins, 4 °C). The cells were then incubated at 37 °C (4 h), fixed, and imaged by confocal microscopy (scale bar = 50 μm). **Figure S11.** Cellular internalization of proteins mediated by cell-surface bound CFAG peptide. Biotinylated CFAG peptide was bound to the cell-surface of primary brain endothelial cells (1 h, 4 °C). The cells were then incubated at 37 °C for 2 h to allow the endocytic removal of CFAG peptide. Avidin-FITC was then bound to the CFAG peptide remaining (retained) on the cell-surface and the cellular internalization of avidin-FITC assessed at various time-points (incubation at 37 °C) by confocal microscopy. Green signal, FITC; blue signal, Hoechst 33342. Scale bar = 50 um. **Figure S12.** UV–vis absorbance spectra of phage library stock and recovered phage population from the first bio-panning step (a). Calibration curve of uv–vis absorbance of phage library (b). **Diagram S1.** Schematic of avidin-FITC binding assays. **Diagram S2.** Schematic of bio-panning procedure to select phage-displayed peptides which bind to (binding population) or are retained on the surface of endothelial cells derived from rat lung, liver and brain. **Diagram S3.** Schematic representation of confocal microscopy visualization of avidin-FITC binding and internalization dynamics into brain endothelial cells. **Table S1.** Synthesized peptide characteristics. **Scheme S1.** General overview of the novel brain delivery strategy generating artificial brain-specific targets (top panel). Strategy to identify molecular tags (peptides) selectively retained on the surface of brain endothelial cells (bottom panel, 1.) Ability of biotinylated molecular tags to act as artificial targets for the intracellular delivery of avidin-FITC proteins into brain endothelial cells (bottom panel, 2.).

## Data Availability

Datafiles have been made available through the repository CORA—Repositori de Dades de Recerca del CSUC (Consorci de Serveis Universitaris de Catalunya).
