# Supplementary material for: Identifying molecular tags selectively retained on the surface of brain endothelial cells to generate artificial targets for therapy delivery
Source: Fluids Barriers CNS. 2023 Dec 6;20:88. doi: 10.1186/s12987-023-00493-6 (PMC10699038; doi:10.1186/s12987-023-00493-6)
Supplement: Supplementary file 1 — Additional file 1: Figure S1. Binding of avidin-FITC to biotin-labelled extracellular protein domains on endothelial cells. The extracellular domain of cell-membrane proteins on primary rat lung, liver or brain endothelial cells was biotinylated by conjugation with cell-impermeable biotin-NHS (20 mins, 4 °C). Biotin labelling was then assessed by quantifying binding of avidin-FITC (30 min, 4 °C) at increasing concentrations. Results are displayed as mean ± SEM of triplicate measurements. Figure S2. Organ accumulation of nanomicelles functionalized with TfR1-targeting ligands. Mice were injected with biotinylated a-TfR1 antibody (25 μg, tail vein injection). After 15 min or 8 h, mice (two separate groups, each n = 4) were injected with avidin-functionalized polymeric nanomicelles (200 μg, tail vein injection). After 16 h, mice were perfused with PBS and nanomicelle biodistribution quantified in organ homogenates (a). Brain targeting ratio at each time-point is calculated by the ratio of nanomicelle uptake (b). Results are displayed as mean ± SEM,*denotes p ≤ 0.05 as determined by a student’s t-test between respective pairs. Figure S3. Ratio of phages recovered from the‘retained population’ to the phages recovered from the ‘binding population’ from the first bio-panning round from each endothelial cell type. Figure S4. Full DNA/amino acid sequence and frequency of analysed individual clones from the third bio-panning round for each endothelial cell type and selection regime. Figure S5. Venn diagram of enriched phage-displayed peptide sequences shared between endothelial phenotypes in both the binding population and the retained population. Figure S6. Avidin-FITC binding to 1°BEC (a, c) or b.END3 (b, d) mediated by CFAG-biotin, VQNP-biotin or control peptide-biotin (100 μM) (a, b), or the transferrin peptide HAIYPRH-biotin (c, d). Figure S7. Avidin-FITC binding to 1°BEC (a) or C6 astrocytes (b) following incubation with biotinylated peptides (100 μM) in serum containin [file 12987_2023_493_MOESM1_ESM.docx]

**Identifying Molecular Tags Selectively Retained on the Surface of Brain Endothelial Cells to Generate Artificial Targets for Therapy Delivery**

Giulia Maria Porro^1^, Italo Lorandi^1^, Xueying Liu^2^, Kazunori Kataoka^2^, Giuseppe Battaglia^1,3^, Daniel Gonzalez-Carter^1*^

^1^ Institute for Bioengineering of Catalonia (IBEC), Barcelona Institute for Science and Technology (BIST), Barcelona 08028, Spain

^2^ Innovation Center of NanoMedicine (iCONM), Kawasaki Institute of Industrial Promotion, Kawasaki 210-0821, Japan

^3^ Institution of Catalonia for Research and Advanced Studies (ICREA), Barcelona 08010, Spain

^*^ Corresponding author: daniel.gonzalezcarter08@alumni.imperial.ac.uk

**Additional file 1**

**Figure S1. Binding of avidin-FITC to biotin-labelled extracellular protein domains on endothelial cells.** The extracellular domain of cell-membrane proteins on primary rat lung, liver or brain endothelial cells was biotinylated by conjugation with cell-impermeable biotin-NHS (20 mins, 4^o^C). Biotin labelling was then assessed by quantifying binding of avidin-FITC (30 mins, 4^o^C) at increasing concentrations. Results are displayed as mean + SEM of triplicate measurements.

**Figure S2.** Organ accumulation of nanomicelles functionalized with TfR1-targeting ligands. Mice were injected with biotinylated a-TfR1 antibody (25 ug, tail vein injection). After 15 min or 8 hrs, mice (two separate groups, each n = 4) were injected with avidin-functionalized polymeric nanomicelles (200 ug, tail vein injection). After 16hr, mice were perfused with PBS and nanomicelle biodistribution quantified in organ homogenates (a). Brain targeting ratio at each time-point is calculated by the ratio of nanomicelle uptake (b). Results are displayed as mean + SEM, * denotes *p* ≤ 0.05 as determined by a student’s t-test between respective pairs.

**Figure S3.** Ratio of phages recovered from the ‘retained population’ to the phages recovered from the ‘binding population’ from the first bio-panning round from each endothelial cell type.

**Figure S4.** Full DNA/amino acid sequence and frequency of analysed individual clones from the third bio-panning round for each endothelial cell type and selection regime.

**Figure S5.** Ven diagram of enriched phage-displayed peptide sequences shared between endothelial phenotypes in both the binding population and the retained population.

**Figure S6.** Avidin-FITC binding to 1^o^BEC (a, c) or b.END3 (b, d) mediated by CFAG-biotin, VQNP-biotin or control peptide-biotin (100 uM) (a, b), or the transferrin peptide HAIYPRH-biotin (c, d).

**Figure S7**. Avidin-FITC binding to 1^o^BEC (a) or C6 astrocytes (b) following incubation with biotinylated peptides (100 uM) in serum containing medium (1hr, 37^o^C) (a) or HBSS (1 hr, 4^o^C) (b), respectively. CFAG-Cy5 binding to b.End3 cells at increasing concentrations (c). Competition of CFAG-Cy5 (3.13 uM) peptide binding to b.End3 cells by unlabelled CFAG (CFAG-biotin) (d).

**Figure S8**. Endothelial targeting of avidin by differential retention of CFAG on the cell surface was assessed by the ratio of avidin-FITC binding between each cell phenotype with time (a, b). Due to the negative value of avidin-FITC RFU at 6hrs for liver EC in (b) (i.e., avidin-FITC binding was slightly lower compared to binding to cells without CFAG treatment), the brain/liver ratio could not be calculated for this time-point. Hence, we have also displayed the liver/brain and lung/brain targeting ratio (c).


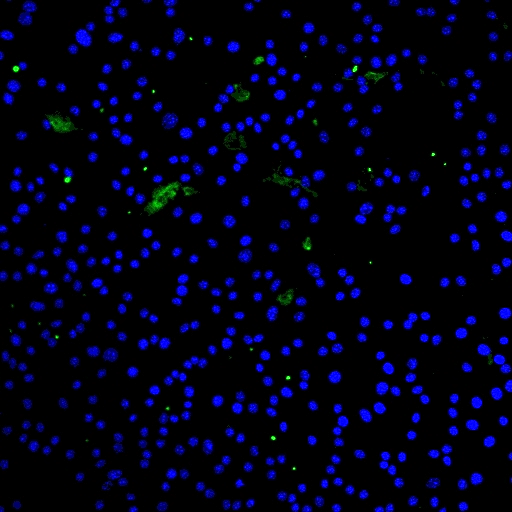

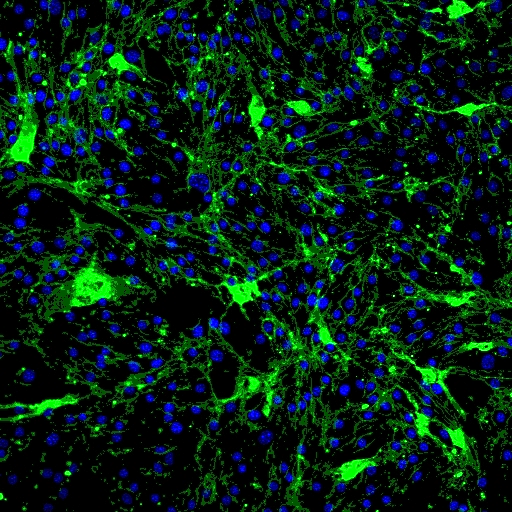


a) 0 uM

b) 50 uM

**Figure S9.** Avidin-FITC binding to b.End3 cells in the absence (PBS treatment, a) or presence (50 uM, b) of biotinylated CFAG peptide.


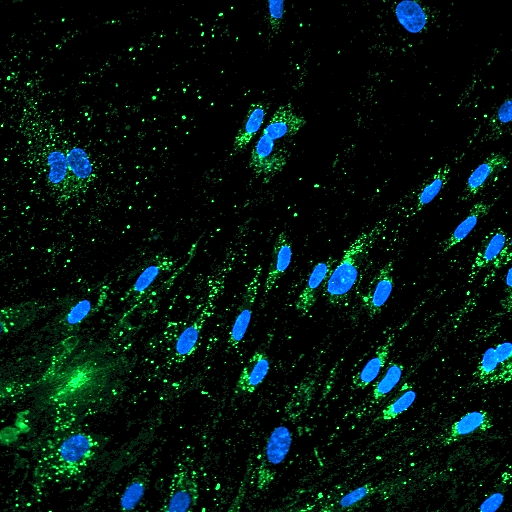


4h


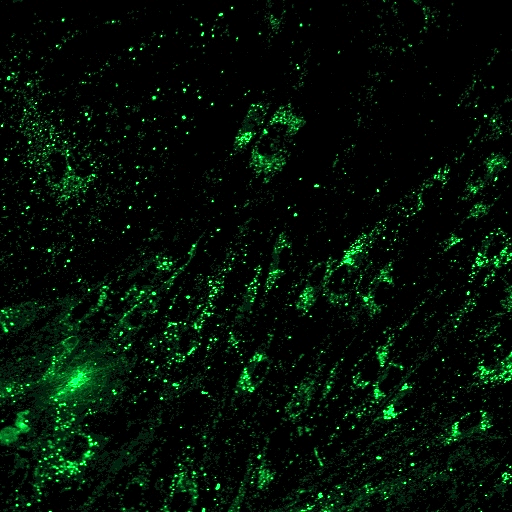

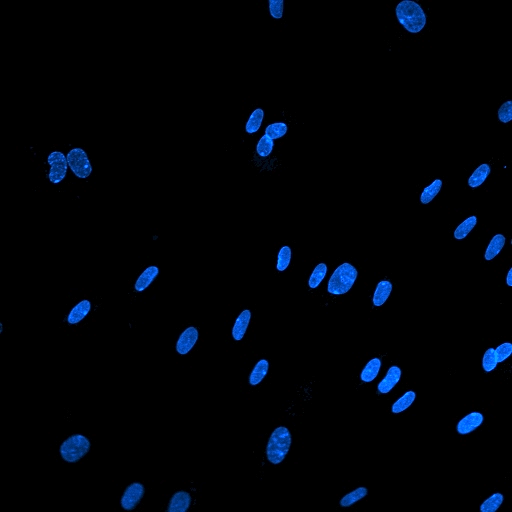


**Figure S10.** Visualization of avidin-FITC internalization into primary brain endothelial cells mediated by CFAG-biotin (50 uM). Cells were treated with CFAG-biotin (1hr, 4^o^C), followed by binding of avidin-FITC (30 mins, 4^o^C). The cells were then incubated at 37^o^C (4 hrs), fixed, and imaged by confocal microscopy (scale bar = 50 um).


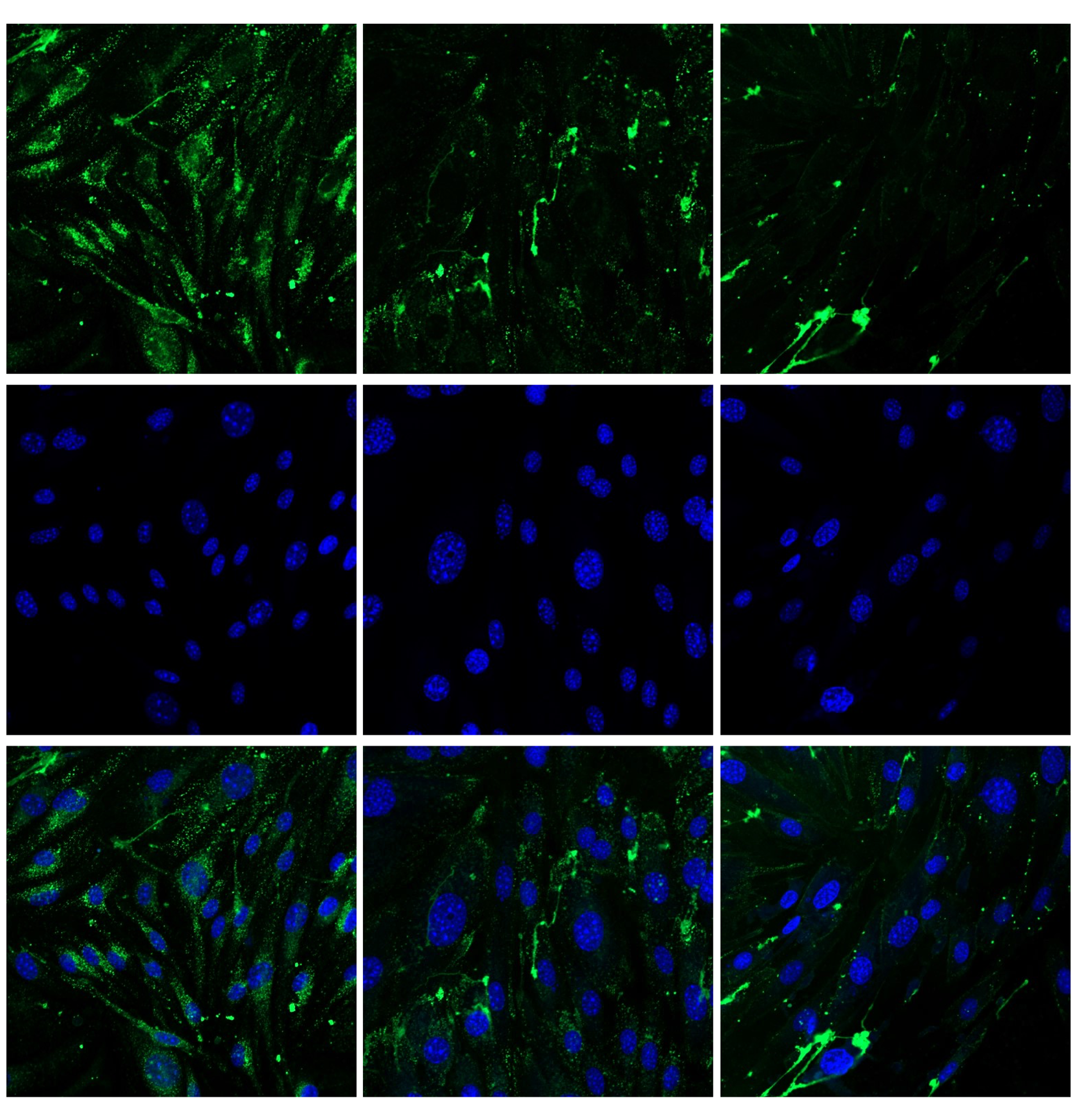


3h

6h

0h

3h

6h

0h

3h

6h

0h

**Figure S11.** Cellular internalization of proteins mediated by cell-surface bound CFAG peptide. Biotinylated CFAG peptide was bound to the cell-surface of primary brain endothelial cells (1 hr, 4^o^C). The cells were then incubated at 37^o^C for 2 hrs to allow the endocytic removal of CFAG peptide. Avidin-FITC was then bound to the CFAG peptide remaining (retained) on the cell-surface and the cellular internalization of avidin-FITC assessed at various time-points (incubation at 37^o^C) by confocal microscopy. Green signal, FITC; blue signal, Hoechst 33342. Scale bar = 50 um.


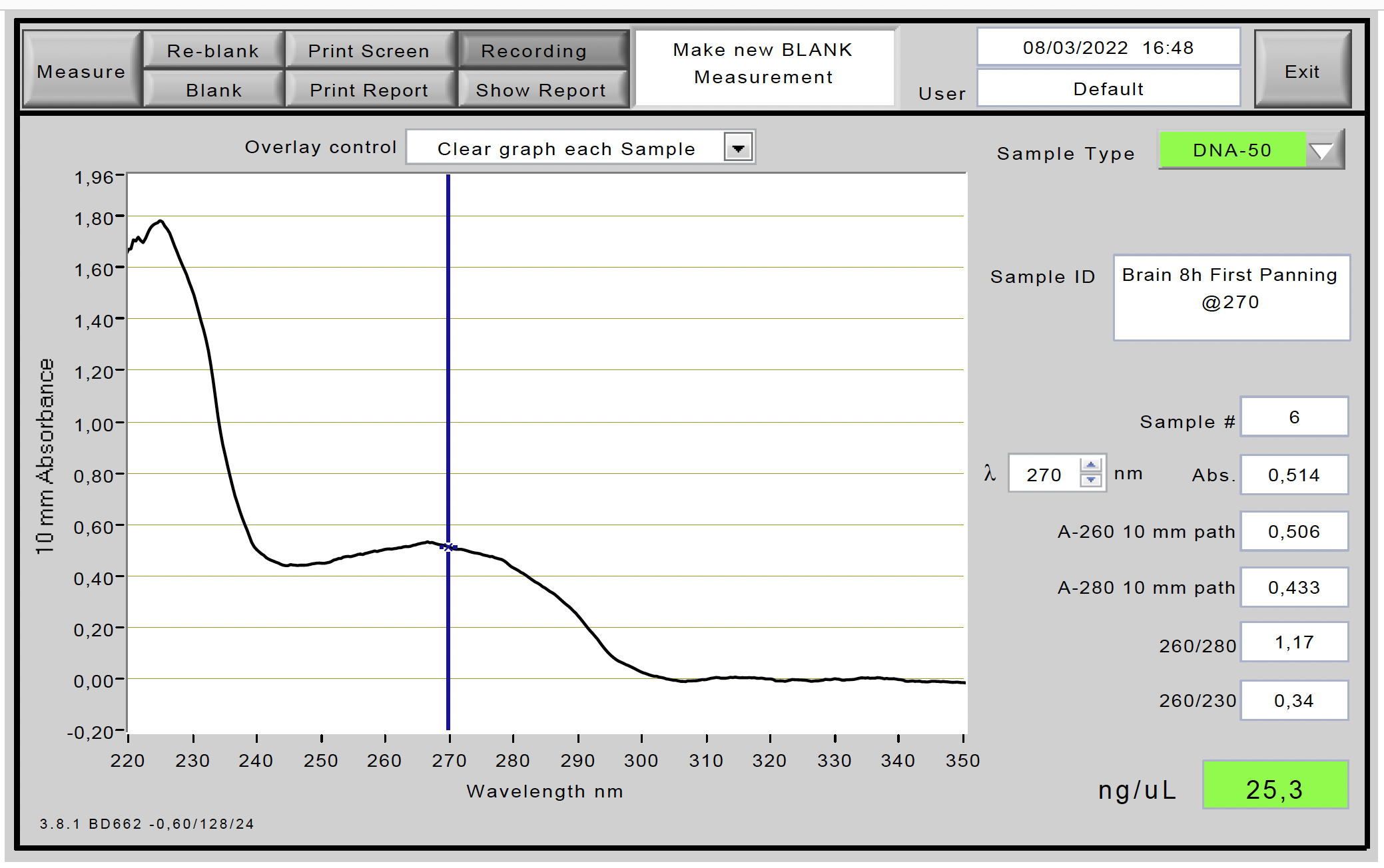

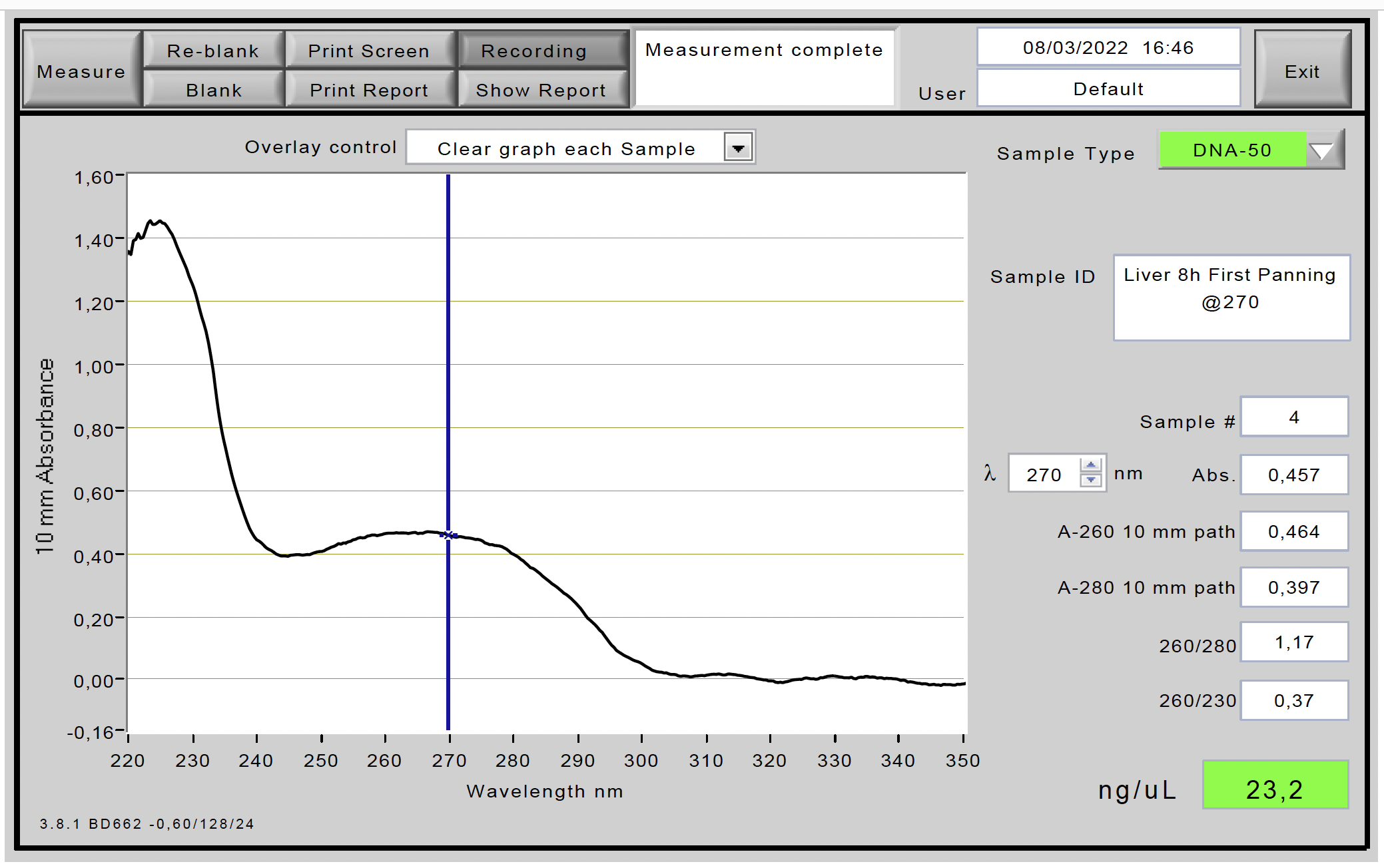

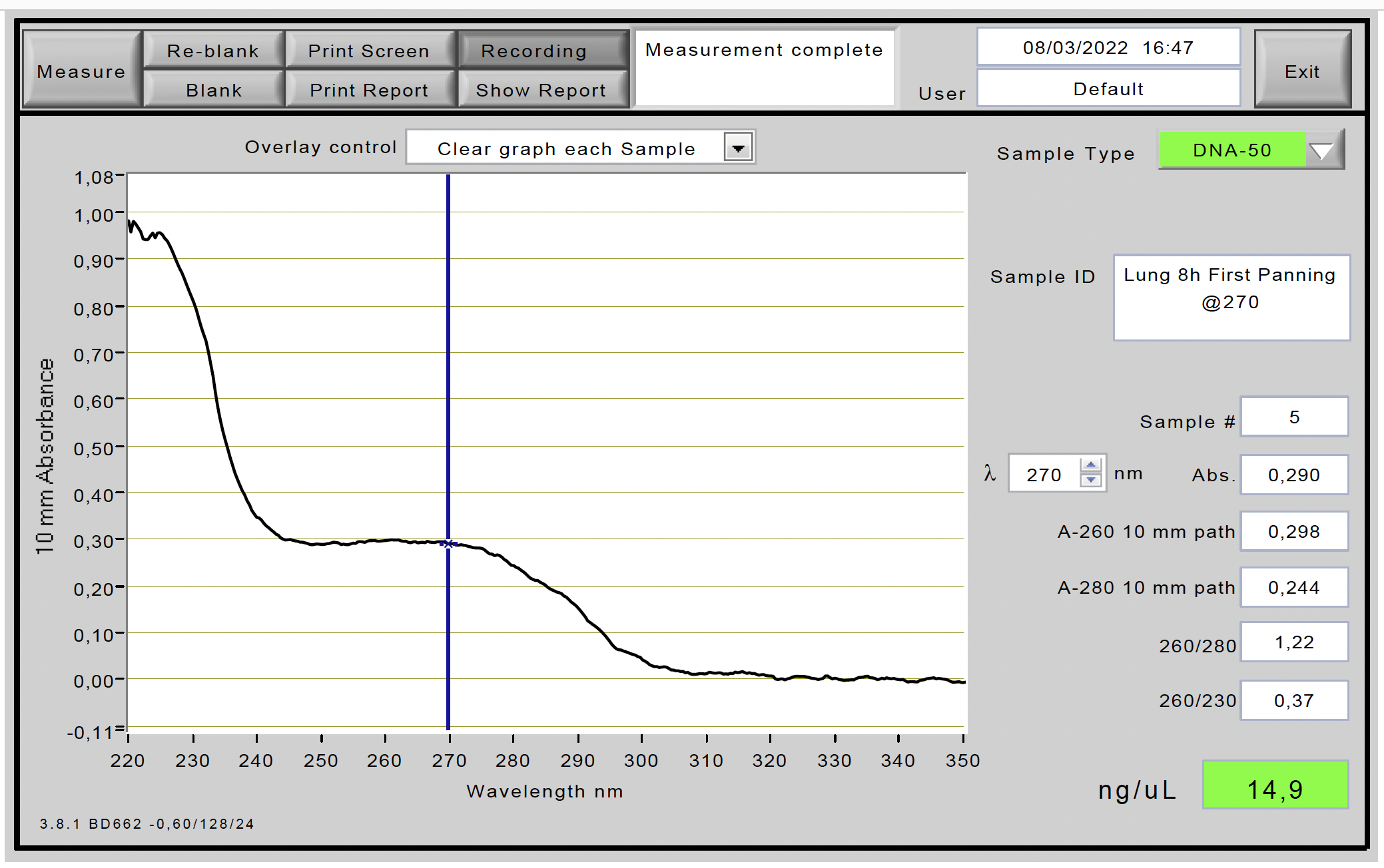

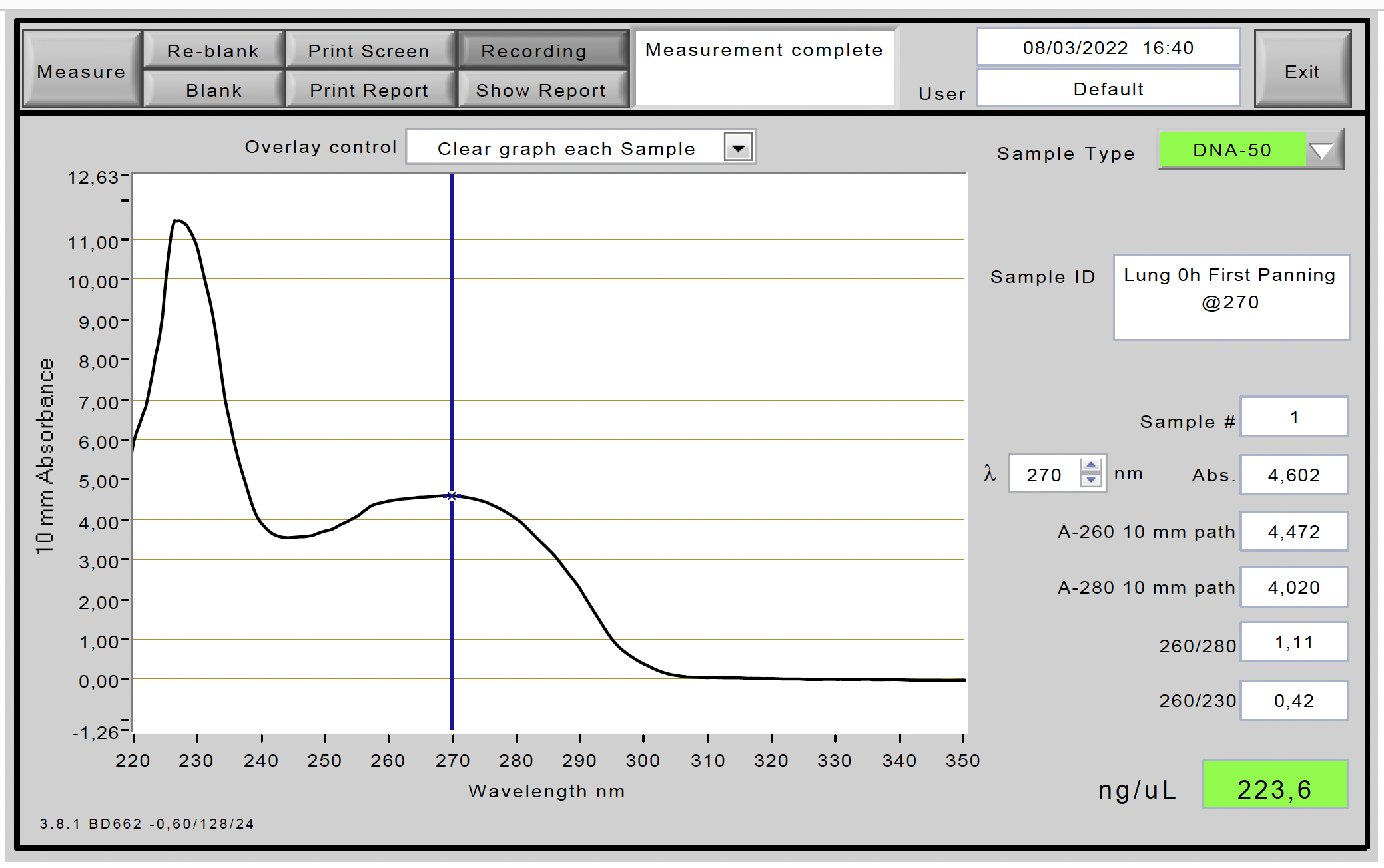

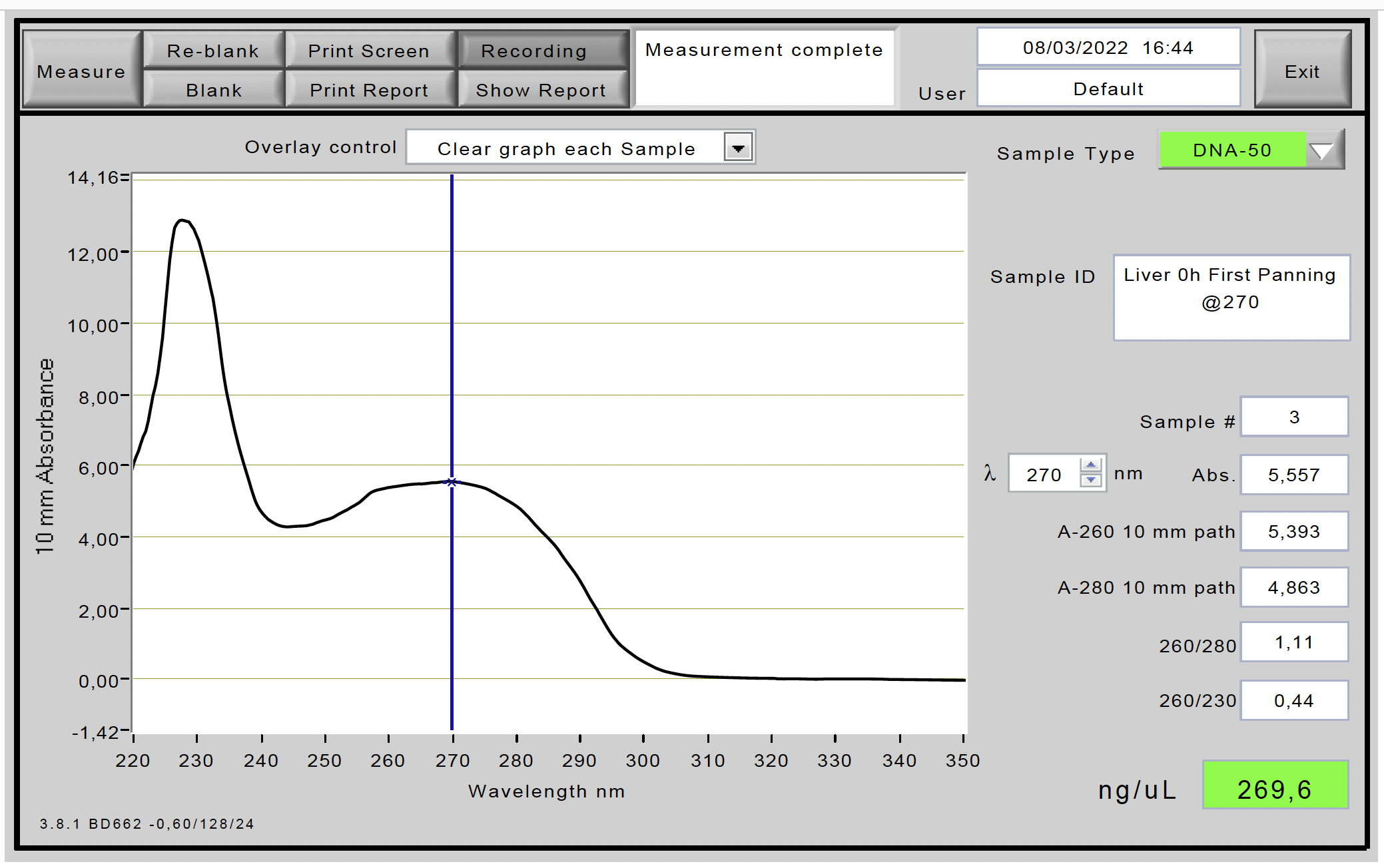

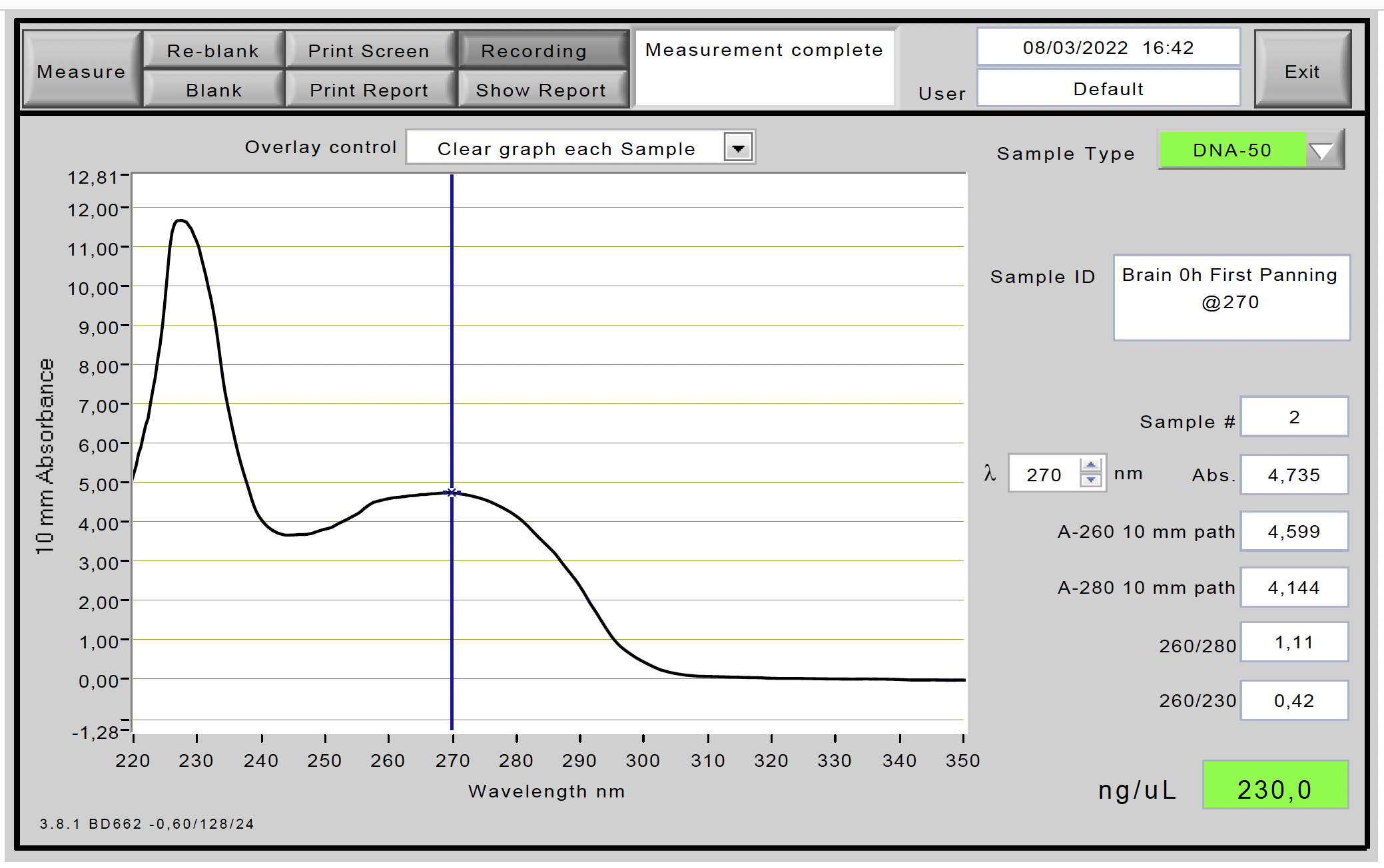

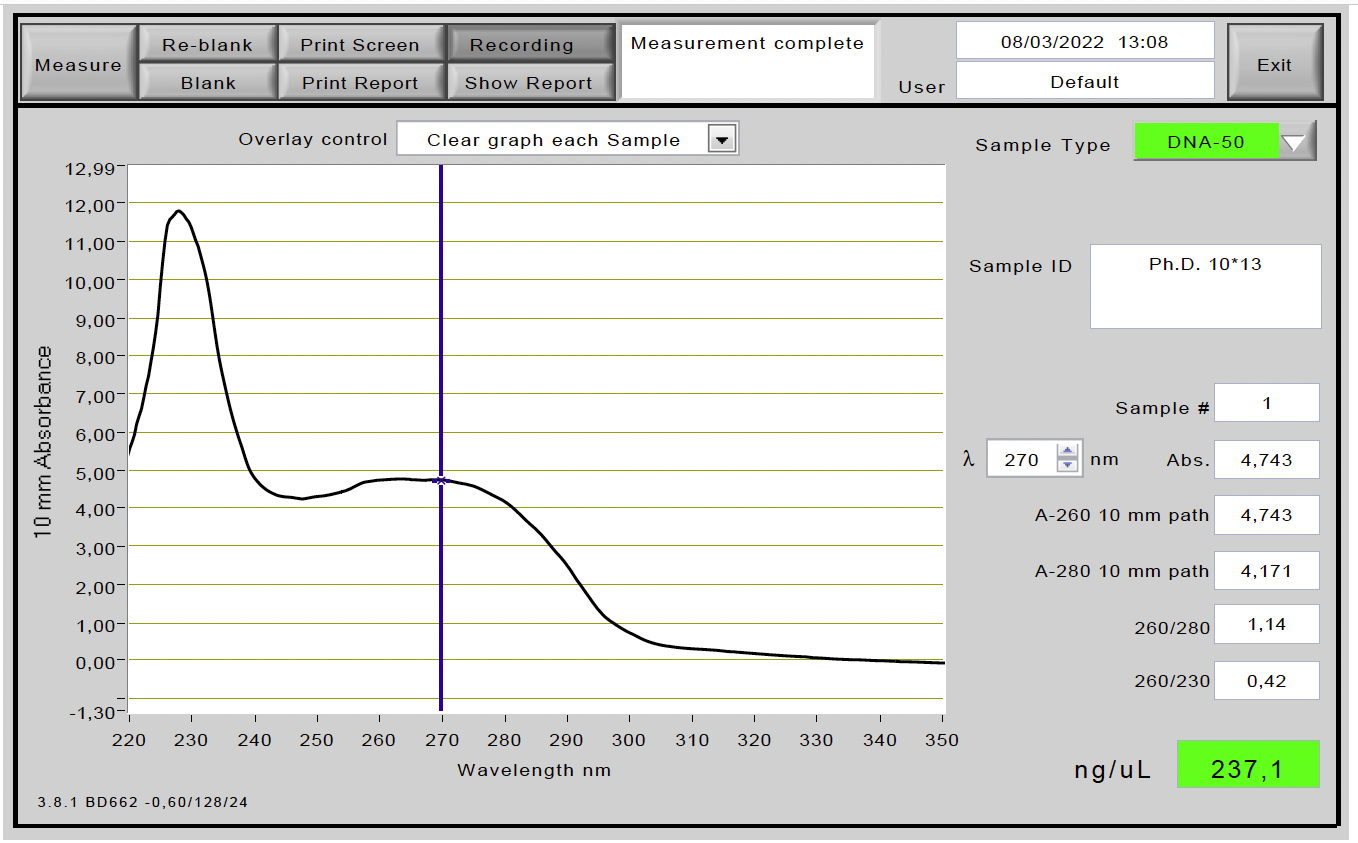

**Figure S12.** UV-vis absorbance spectra of phage library stock and recovered phage population from the first bio-panning step (a). Calibration curve of uv-vis absorbance of phage library (b).

**Diagram S1.** Schematic of avidin-FITC binding assays.


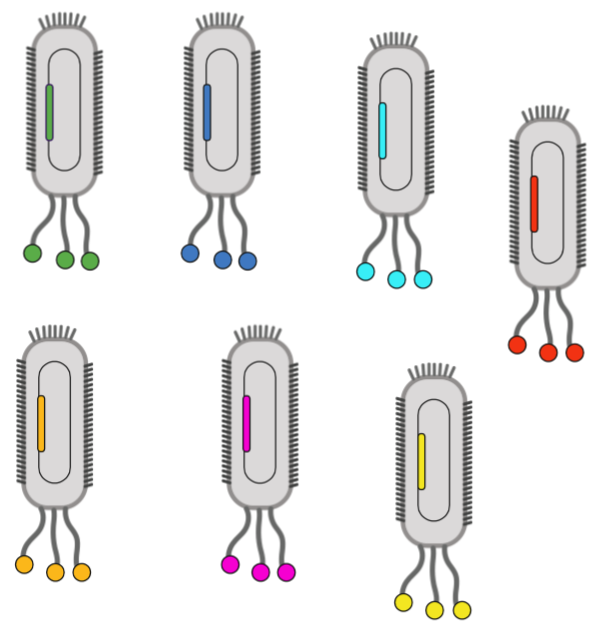

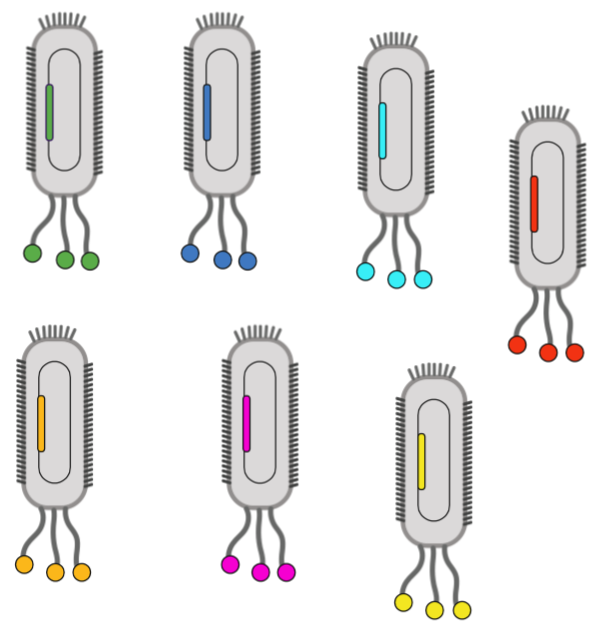

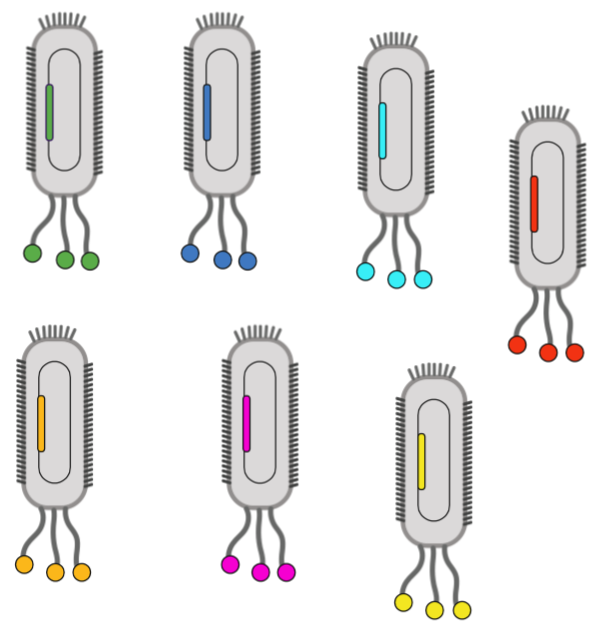

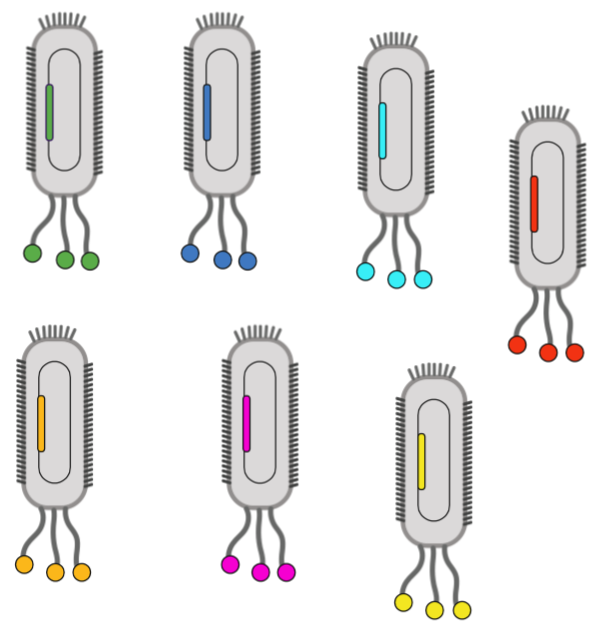


(8 hr, 37^o^C)

Endocytic removal

**Binding Population**

**Retained Population**

Wash

Bind


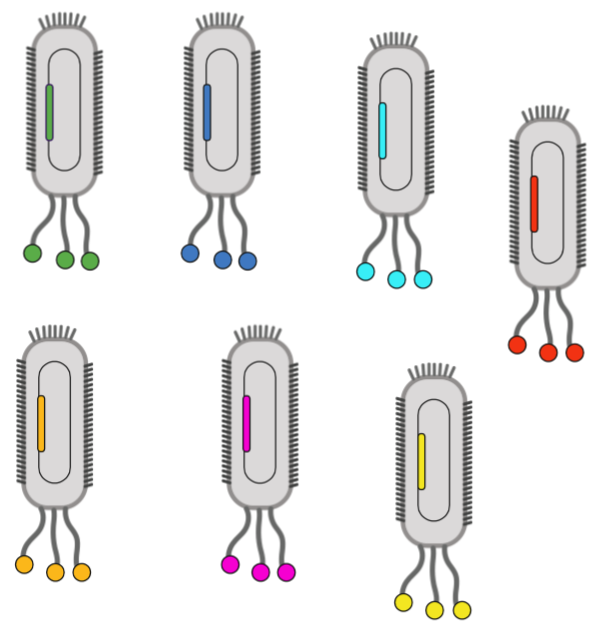

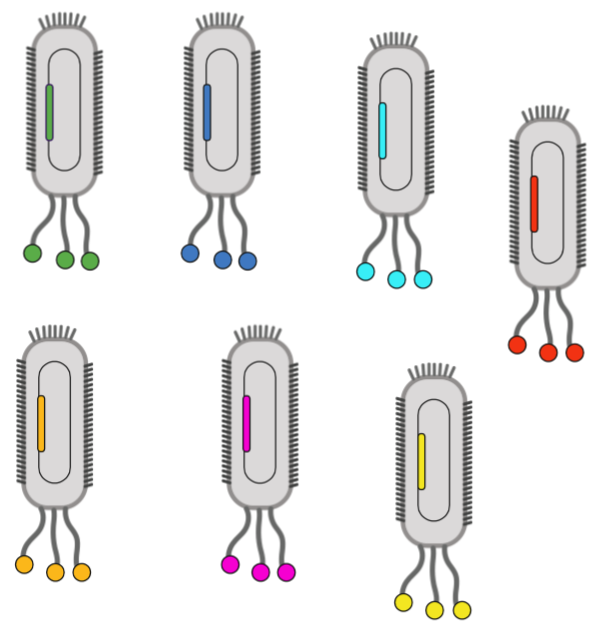

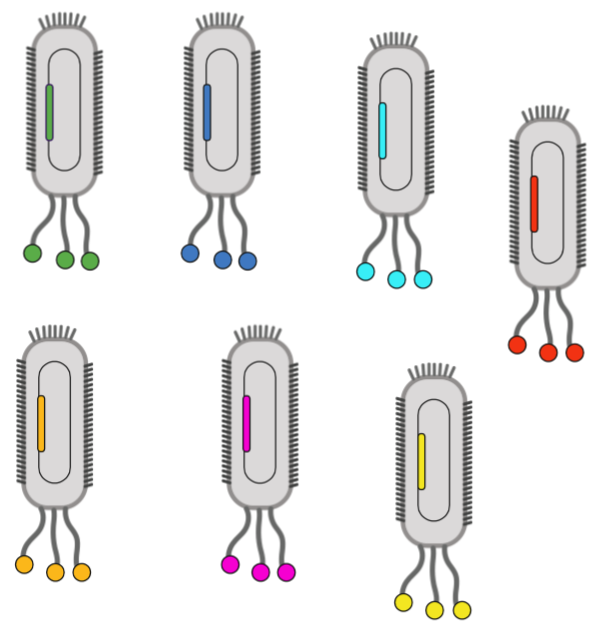

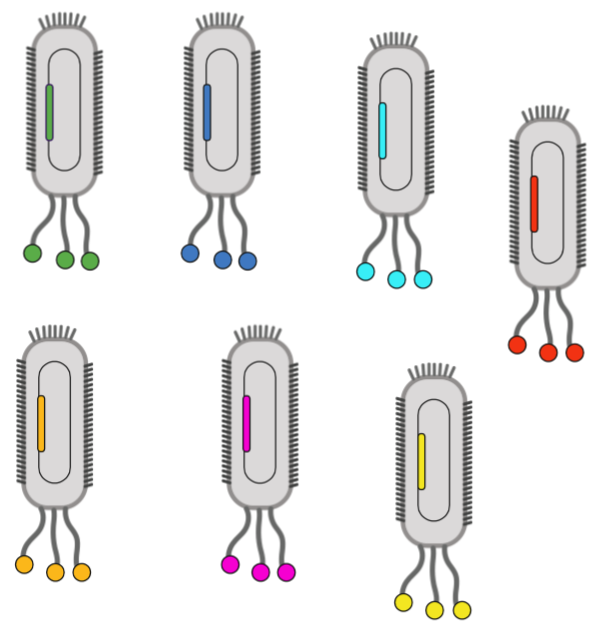

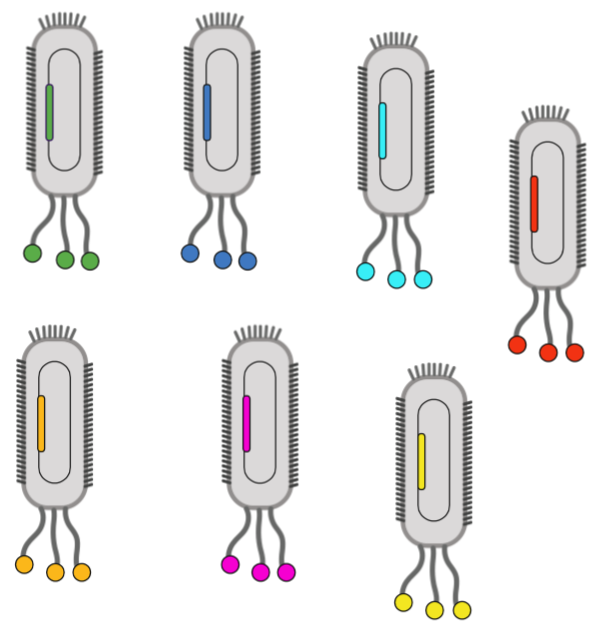

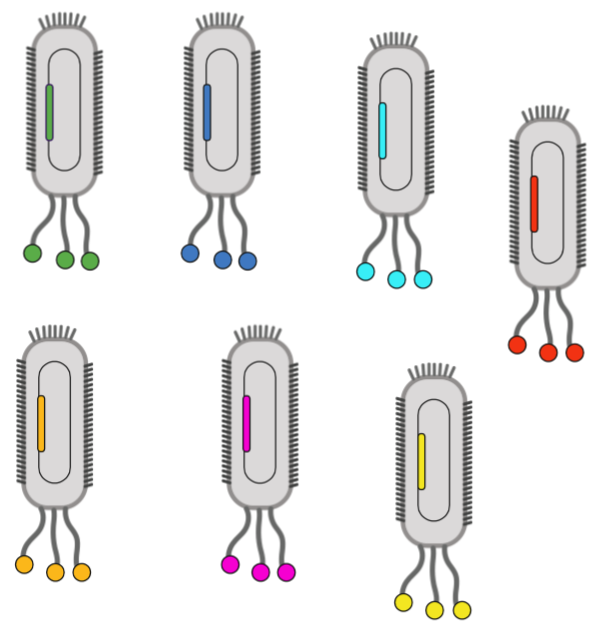

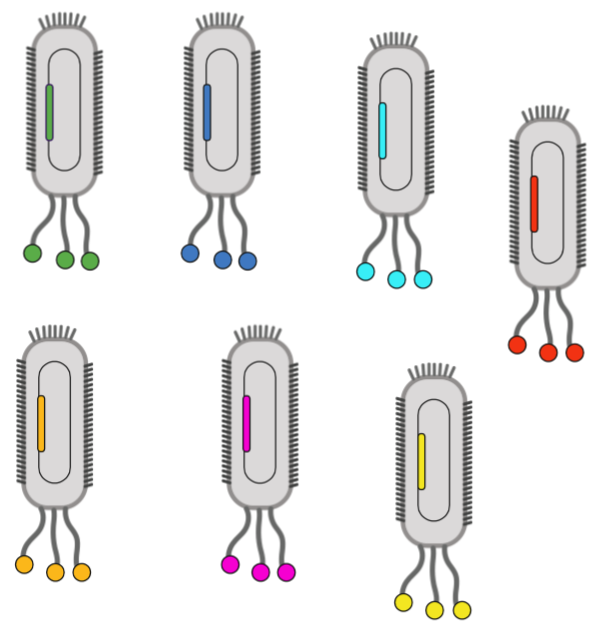

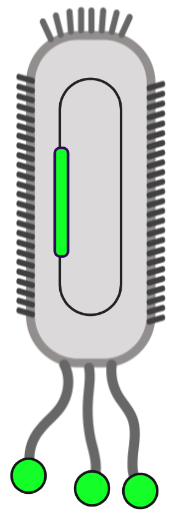

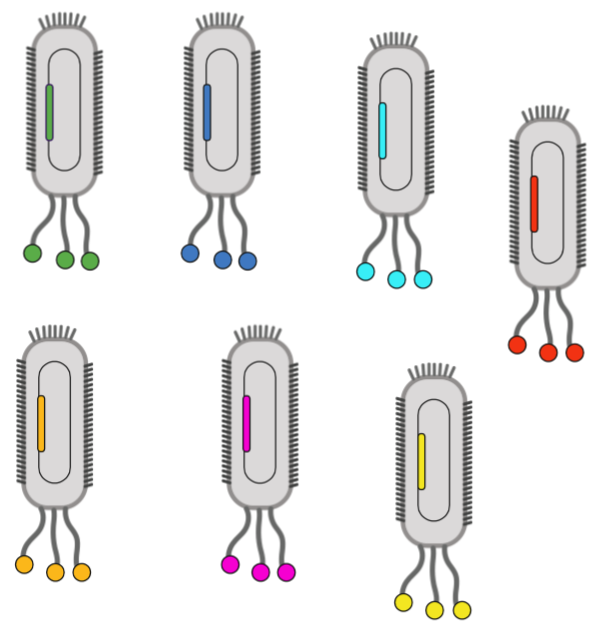

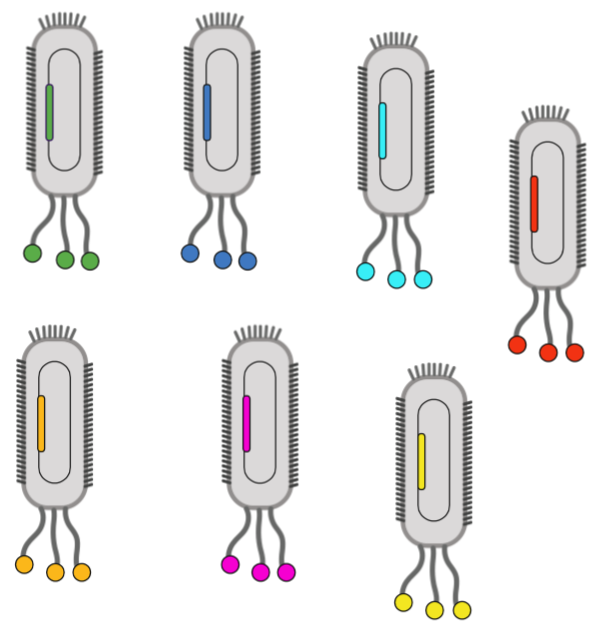

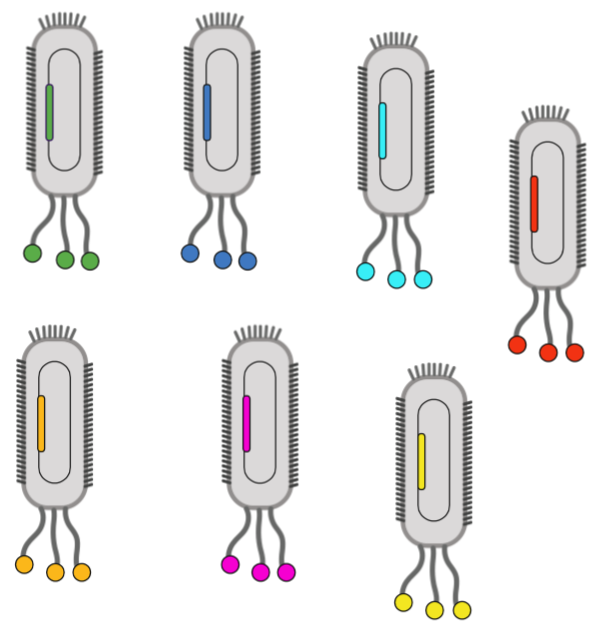

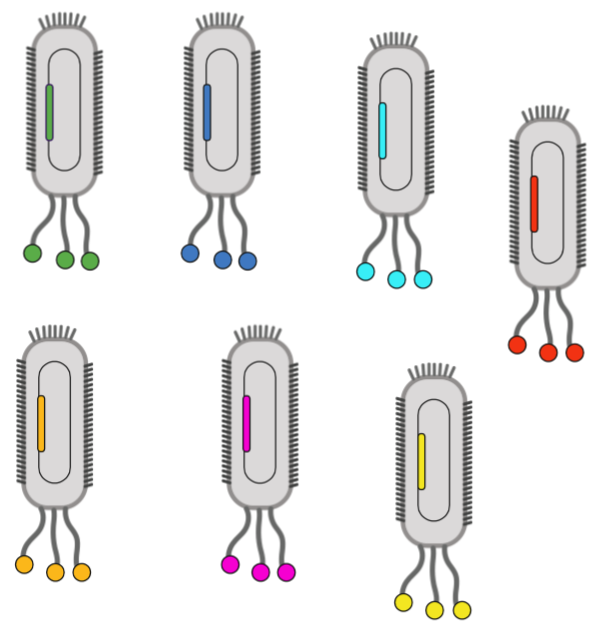

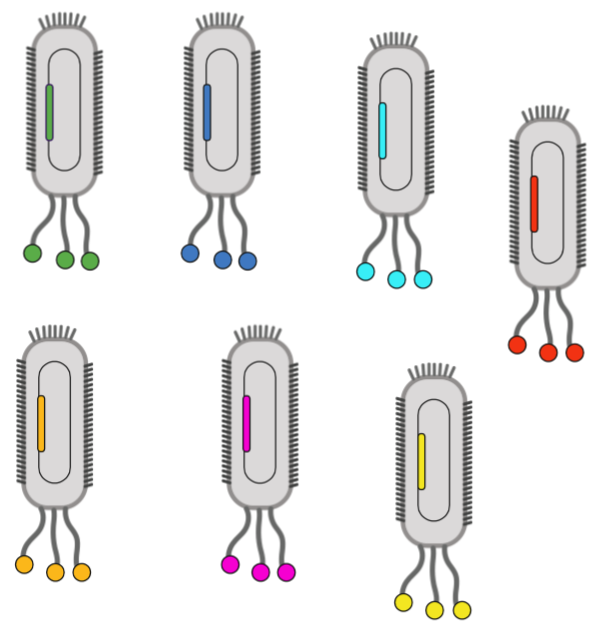

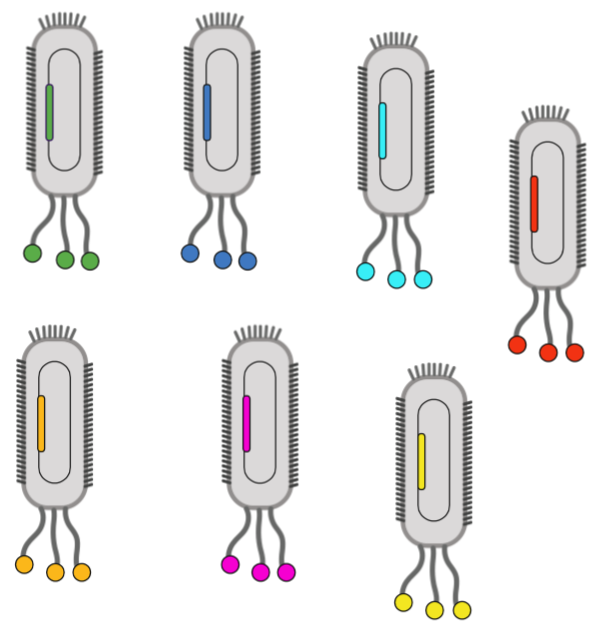

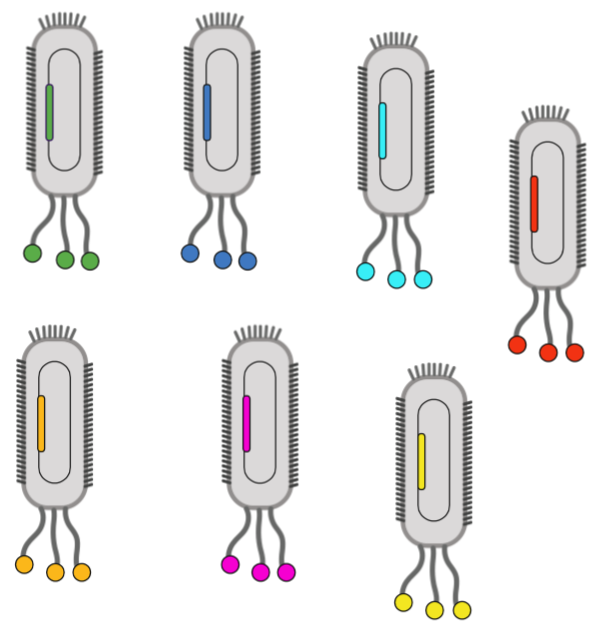

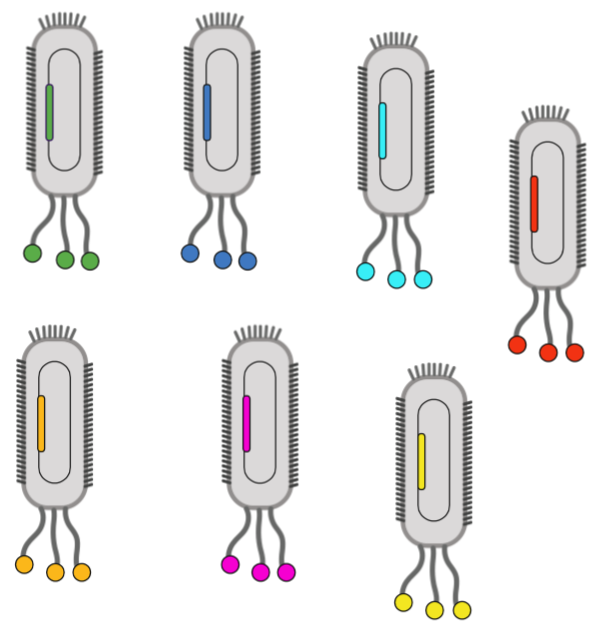

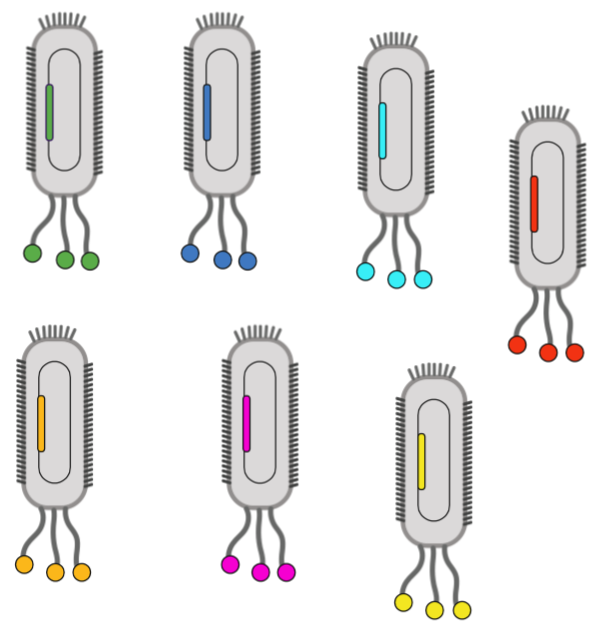

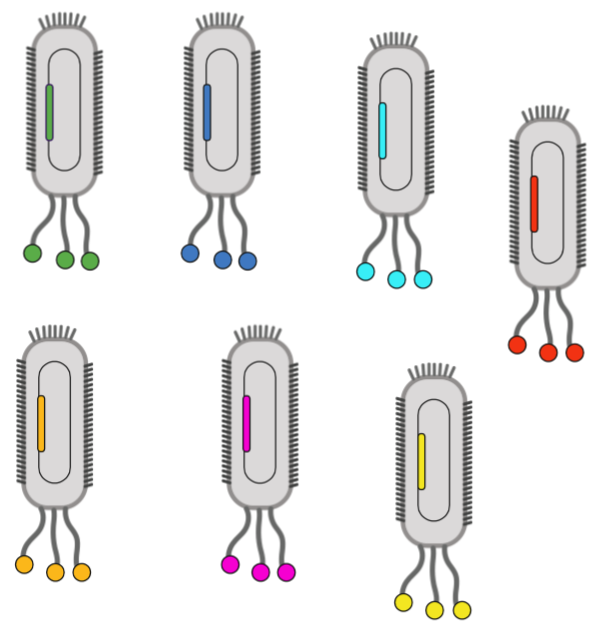

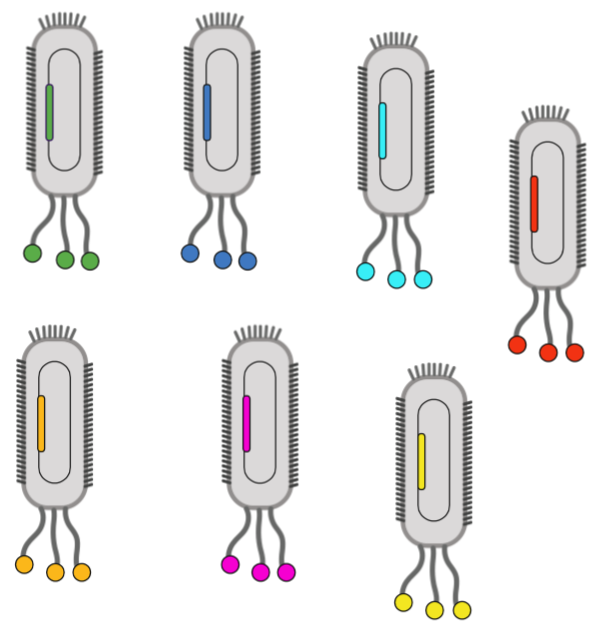

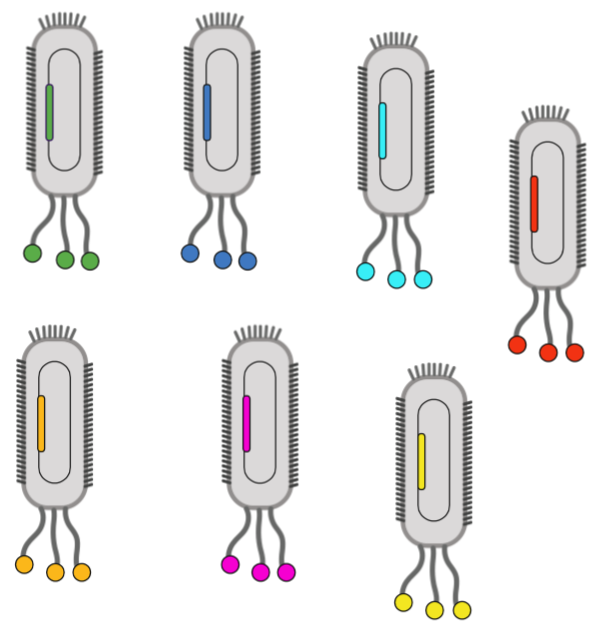

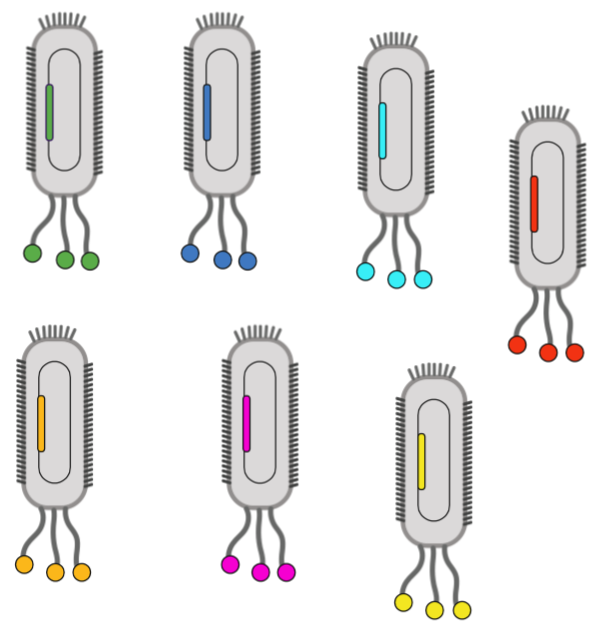


Amplify

Amplify

Endothelial cell

Repeat binding (x2)

*Recover*

*Recover*


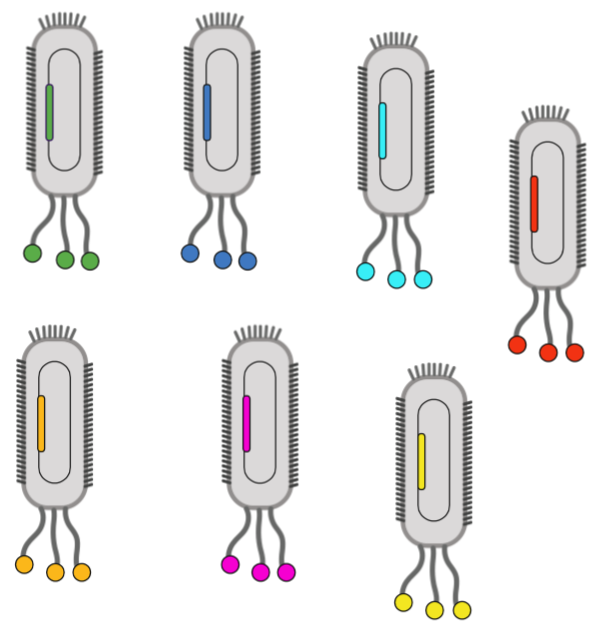

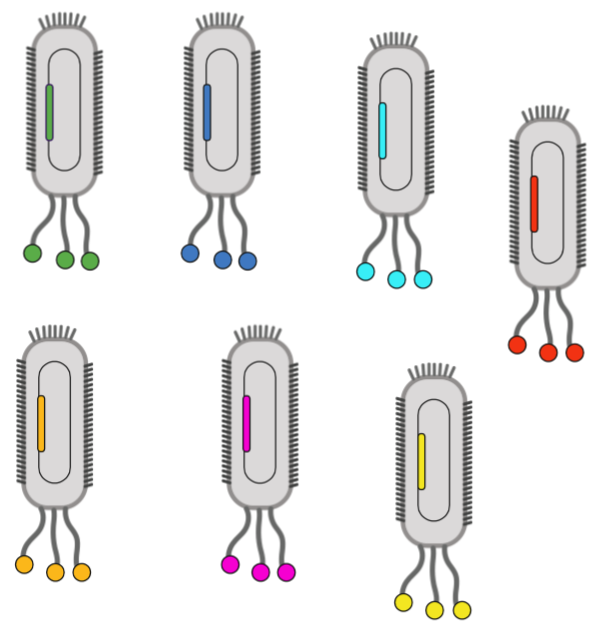

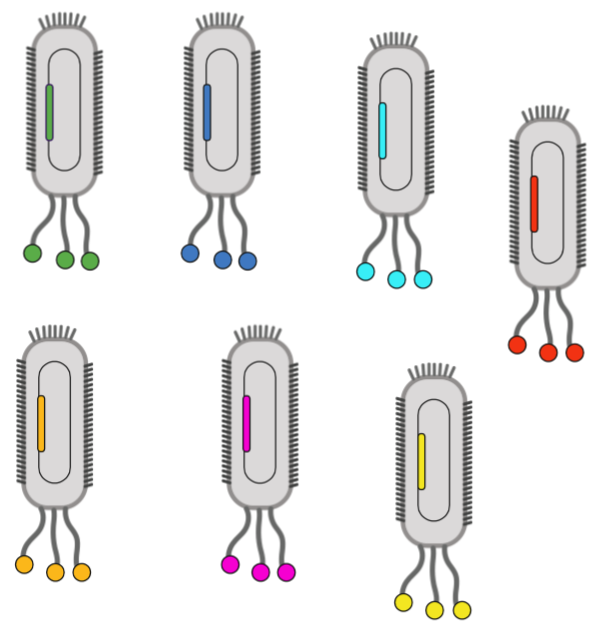

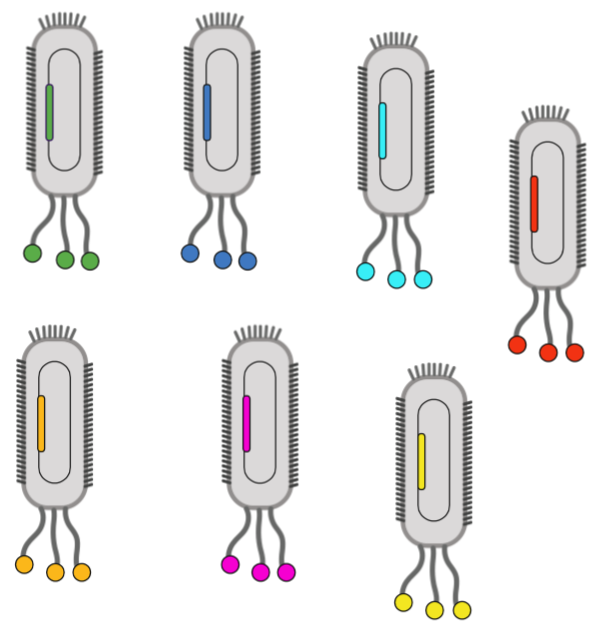


*(Brain, Liver or Lung)*

Repeat binding (x2)

**M13 phage library**

**Diagram S2.** Schematic of bio-panning procedure to select phage-displayed peptides which bind to (binding population) or are retained on the surface of endothelial cells derived from rat lung, liver and brain.

**Diagram S3.** Schematic representation of confocal microscopy visualization of avidin-FITC binding and internalization dynamics into brain endothelial cells.

**Table S1. Synthesized peptide characteristics**

**Scheme S1.** General overview of the novel brain delivery strategy generating artificial brain-specific targets (top panel). Strategy to identify molecular tags (peptides) selectively retained on the surface of brain endothelial cells (bottom panel, 1.) Ability of biotinylated molecular tags to act as artificial targets for the intracellular delivery of avidin-FITC proteins into brain endothelial cells (bottom panel, 2.).
